# Pervasive Selection for Clinically Relevant Resistance and Media Adaptive Mutations at Very Low Antibiotic Concentrations

**DOI:** 10.1093/molbev/msad010

**Published:** 2023-01-11

**Authors:** Catia Pereira, Omar M Warsi, Dan I Andersson

**Affiliations:** Department of Medical Biochemistry and Microbiology, Uppsala University, Uppsala, Sweden; Department of Medical Biochemistry and Microbiology, Uppsala University, Uppsala, Sweden; Department of Medical Biochemistry and Microbiology, Uppsala University, Uppsala, Sweden

**Keywords:** antibiotic resistance, selection, *Escherichia coli*, media adaptation, subMIC, fitness

## Abstract

Experimental evolution studies have shown that weak antibiotic selective pressures (i.e., when the antibiotic concentrations are far below the minimum inhibitory concentration, MIC) can select resistant mutants, raising several unanswered questions. First, what are the lowest antibiotic concentrations at which selection for de novo resistance mutations can occur? Second, with weak antibiotic selections, which other types of adaptive mutations unrelated to the antibiotic selective pressure are concurrently enriched? Third, are the mutations selected under laboratory settings at subMIC also observed in clinical isolates? We addressed these questions using *Escherichia coli* populations evolving at subMICs in the presence of either of four clinically used antibiotics: fosfomycin, nitrofurantoin, tetracycline, and ciprofloxacin. Antibiotic resistance evolution was investigated at concentrations ranging from 1/4th to 1/2000th of the MIC of the susceptible strain (MIC_susceptible_). Our results show that evolution was rapid across all the antibiotics tested, and selection for fosfomycin- and nitrofurantoin-resistant mutants was observed at a concentration as low as 1/2000th of MIC_susceptible_. Several of the evolved resistant mutants showed increased growth yield and exponential growth rates, and outcompeted the susceptible ancestral strain in the absence of antibiotics as well, suggesting that adaptation to the growth environment occurred in parallel with the selection for resistance. Genomic analysis of the resistant mutants showed that several of the mutations selected under these conditions are also found in clinical isolates, demonstrating that experimental evolution at very low antibiotic levels can help in identifying novel mutations that contribute to bacterial adaptation during subMIC exposure in real-life settings.

## Introduction

Preexisting and de novo-generated resistant mutants can be selected at very low antibiotic concentrations that are well below the minimum inhibitory concentration (subMIC) of a given antibiotic (Davies et al. 2006; Olofsson et al. 2007; Gullberg et al. 2011, 2014; Westhoff et al. 2017; Wistrand-Yuen et al. 2018; Sanz-García et al. 2020; Stanton et al. 2020; Gu et al. 2021). These results imply that the selection of antibiotic resistance is likely to also occur in many environments where antibiotics are present at low concentrations due to anthropogenic pollution, for example in soil, surface water, and wastewater treatment plants, as well as in certain tissues/compartments in antibiotic-treated humans and animals (Baquero and Negri 1997; Kümmerer and Henninger 2003; Berge et al. 2005; Chee-Sanford et al. 2009; Heuer et al. 2011; McManus 2014; Gao et al. 2015; Shenje et al. 2015; Thabit et al. 2019). Although these studies have demonstrated that subMIC antibiotic levels can indeed select for resistance, the potential for the emergence of *clinically relevant* mutations under such selection regimes remains largely unclear. A few studies have shown the emergence of resistance mutations that are found in clinical isolates under subMIC antibiotic exposure, for example, for fluoroquinolone (Sanz-García et al. 2022) and streptomycin (Wistrand-Yuen et al. 2018) subMIC levels selected for mutations in the fluoroquinolone target DNA gyrase (*gyrA*) and the *gidB* gene (encodes an enzyme that modifies the ribosome), respectively. These limited number of studies accentuate the need to systematically determine if clinically relevant resistance mutations are selected under subMIC exposure and to examine the nature of these mutations.

Furthermore, since selection for resistance at antibiotic levels much below the MIC is weak, other concurrent selective pressures present in these environments, such as adaptation to the specific growth conditions (energy/carbon sources, temperature, and pH), as well as compensatory evolution to reduce the fitness costs of any emerging resistance mutations (Björkman et al. 2000; Moura de Sousa et al. 2015; Gifford et al. 2016), will also influence the evolutionary trajectory. These considerations raise questions regarding the relative importance of *bona fide* resistance mutations and media adaptive mutations, respectively, at subMIC levels and also whether certain mutations can concomitantly increase resistance and confer media adaptation. Precedent for the existence of the latter type of mutations comes from two previous studies which showed that a small fraction of mutants selected for adaptation to the specific growth media, in the absence of any antibiotic exposure, coincidentally also showed increased resistance to some antibiotics (Knöppel et al. 2017; Lamrabet et al. 2019). These results suggest that there exist a pool of mutations that pleiotropically can cause both increased resistance and media adaptation, and thus, they could potentially play a role during subMIC evolution. Regarding compensatory evolution at subMIC, to the best of our knowledge, only a single study utilizing *Streptomyces coelicolor* and subMIC exposure of streptomycin (Westhoff et al. 2017) has suggested the role of compensatory mutations in the selection of resistant mutants.

For this study, we examined antibiotics that are clinically relevant and are used to treat human and animal infections caused by *E. coli*, including nitrofurantoin, ciprofloxacin, and fosfomycin (Wagenlehner et al. 2013; Pérez et al. 2014; Huttner et al. 2015; Papich 2017; Huttner et al. 2018; Kot 2019; Drekonja et al. 2021; Leuin et al. 2021). We also included the antibiotic tetracycline, which is used for other types of infections (Roberts 2003) and is commonly found at low concentrations in many natural environments (Liu et al. 2018; Song et al. 2021; Wang et al. 2021). For each of these antibiotics, the selection was carried out over a wide-range of subMIC antibiotic levels to determine the minimal selective concentration (MSCs), where MSC is defined as the lowest antibiotic concentration where the fitness cost of the resistance is balanced by the antibiotic-conferred selection for the resistant mutant (Gullberg et al. 2011). Individual resistant mutants were characterized with regard to their resistance level, relative fitness (in single batch culture and by competitions), and cross-resistance to other antibiotics, and were whole-genome sequenced to identify the genes and mutations that conferred increased resistance and/or growth-media adaptation.

## Results

### Selection of Resistant Mutants at subMIC of Antibiotics

**Fig. 1.**
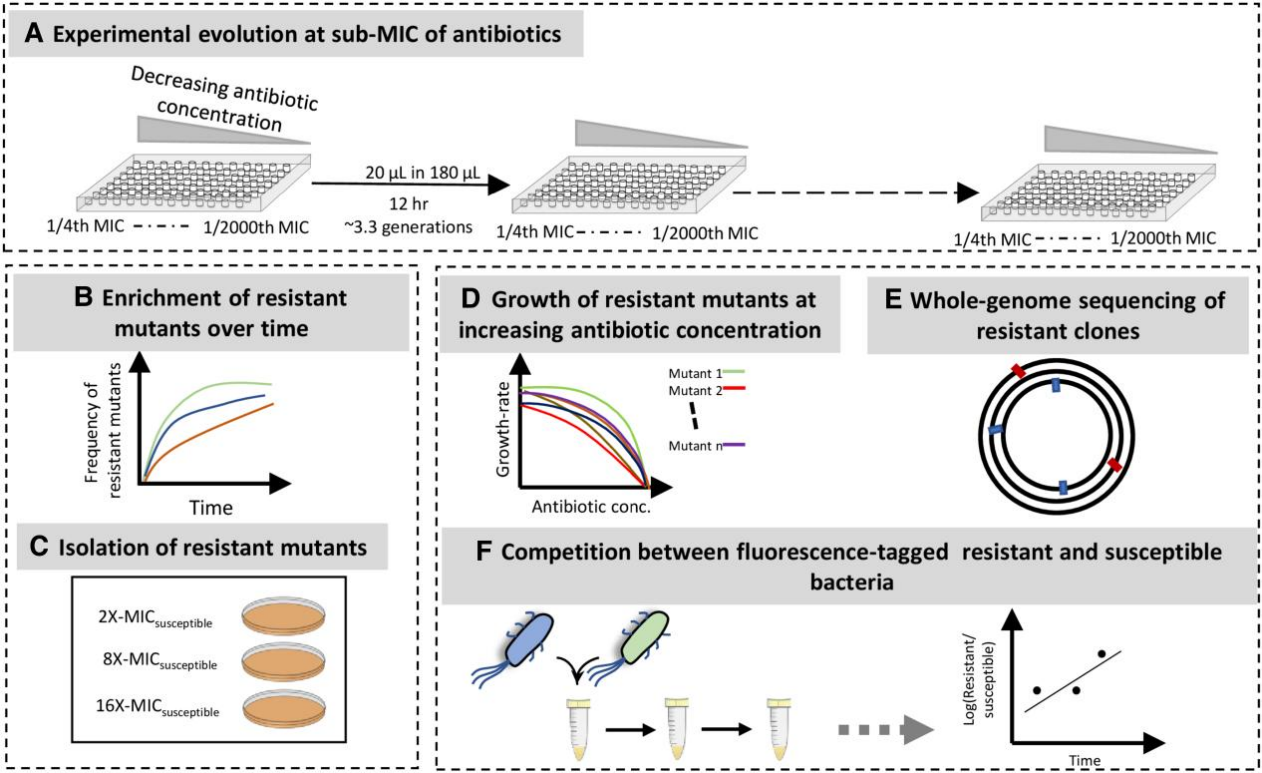
Selection of resistant bacteria at the subMICs of four antibiotics. (*A*) The antibiotic concentrations used in the selection experiments ranged from 1/4th to 1/2000th MIC_susceptible_. Eight lineages were used for each concentration of a given antibiotic, and the experiment was carried out by transferring 20 μl of overnight-grown cultures into 180 μl of fresh media every 12 h. (*B*, *C*) The frequency of resistant bacteria was measured over the course of the experiment by plating approximately 10^6^ cells/lineage on agar plates containing antibiotic concentrations of 2 × -, 4 × -, 8 × -, and 16 × - MIC_susceptible_. (*D*–*F*) Resistant mutants were characterized by measuring the growth rates at different antibiotic concentrations, by identifying adaptive mutations using whole-genome sequencing, and by determining relative fitness using competition assays between fluorescently marked susceptible and resistant strains.

We serially passaged eight lineages of *E. coli* K-12 MG1655 at different subMIC antibiotic concentrations of fosfomycin, tetracycline, nitrofurantoin, and ciprofloxacin to determine the lowest antibiotic concentrations that would result in the selection of resistant mutants (fig. 1). As a control, bacteria were also serially passaged in the absence of antibiotics and under these conditions, no increase in resistance was observed. The antibiotic concentrations used in our experiments ranged from 1/4th of MIC_susceptible_ to 1/2000th of MIC_susceptible_ (where MIC_susceptible_ refers to the MIC of the susceptible *E. coli* K-12 strain for each antibiotic). After every ∼30 generations of serial passage, approximately 10^6^ bacterial cells were plated from each lineage on plates containing antibiotic concentrations of 2 × -, 4 × -, 8 × -, and 16 × MIC_susceptible_ to determine the frequency of resistant mutants in the evolving population. For all the antibiotics tested, we observed an increase in the frequency of resistant mutants over the course of the experiment, although the rate of increase and the lowest concentration at which selection for resistant mutants observed is antibiotic-specific (supplementary fig. S1, Supplementary Material online). For example, fosfomycin selection occurred rapidly and fixation of high-level resistant mutants was observed at concentrations of 1/20th of MIC_susceptible_ at ∼30 generations, while an increase in the frequency of resistant mutants continued to occur even at concentrations below it, although at a slower rate. Despite this difference in rates, the selection of high-level fosfomycin-resistant mutants (ability to grow at an antibiotic concentration of 16 × MIC_susceptible_) was observed at all the different subMICs of fosfomycin, although they only reached fixation at subMICs of 1/50th of MIC_susceptible_ and above. On the other hand, the selection of resistant mutants for ciprofloxacin was observed only at concentrations of 1/4th of MIC_susceptible_ and 1/10th of MIC_susceptible_ after ∼200 generations of cycling, with high-level resistant mutants (ability to grow at an antibiotic concentration of 8 × MIC_susceptible_) only being selected at 1/4th of MIC_susceptible_. For the remaining two antibiotics, the rate of enrichment of resistant mutants ranges between these two extremes (supplementary fig. S1, Supplementary Material online). Thus for tetracycline, high-level resistant mutants (ability to grow at an antibiotic concentration of 16 × MIC_susceptible_) were observed at subMICs of 1/50th of MIC_susceptible_ and above, but were only fixed in the population at concentrations of 1/4th of MIC_susceptible_, while resistant mutants that could grow at 4 × MIC_susceptible_ were observed at all the subMICs tested, and reached fixation at concentrations of 1/200th of MIC_susceptible_ and above. For nitrofurantoin, mutants that could grow at concentrations of 16 × MIC_susceptible_ were observed only at 1/4th of MIC_susceptible_, but did not reach fixation during the course of the experiment, while the mutants that could grow at 4 × MIC_susceptible_ were observed at all the subMICs tested.

### Minimal Selective Concentrations for Different Antibiotics and Resistance Levels of Mutants

**Fig. 2.**
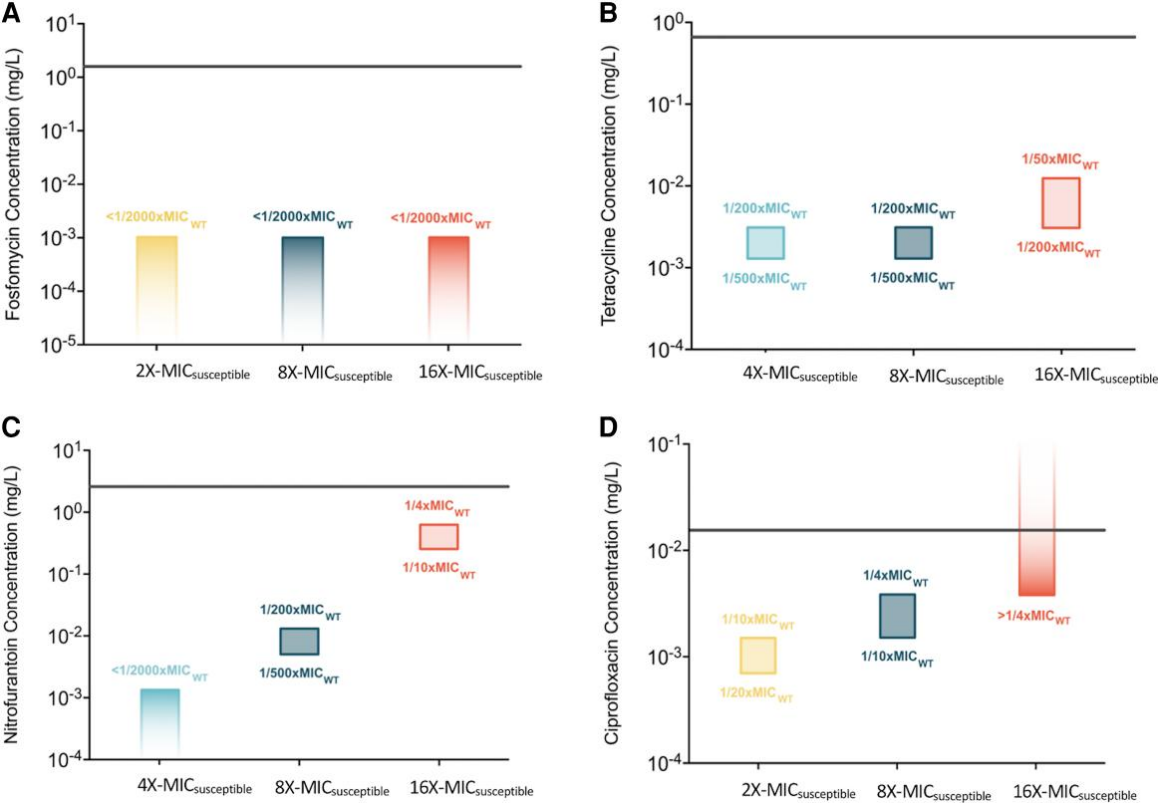
Minimal selective concentrations (MSCs) for resistant mutants. Minimal selective concentrations for mutants with different levels of resistance toward the antibiotics (*A*) fosfomycin, (*B*) tetracycline, (*C*) nitrofurantoin, and (*D*) ciprofloxacin. In cases where the emergence of resistant mutants was observed at a given concentration and was not observed at the next lower concentration, MSC is shown as a range between these two concentrations. Screening of mutants was done by plating evolving populations at different antibiotic concentrations ranging from 2 × - MIC_susceptible_ to 16 × - MIC_susceptible_. The concentrations used for screening the mutants are shown along the x-axis in each plot, and the y-axis shows absolute antibiotic concentrations (log-scale). The MSCs (in relation to the MIC of the susceptible strain) are indicated for each level of resistance. The MIC for each antibiotic, as determined by broth microdilution assay, is represented by the horizontal line.

**Fig. 3.**
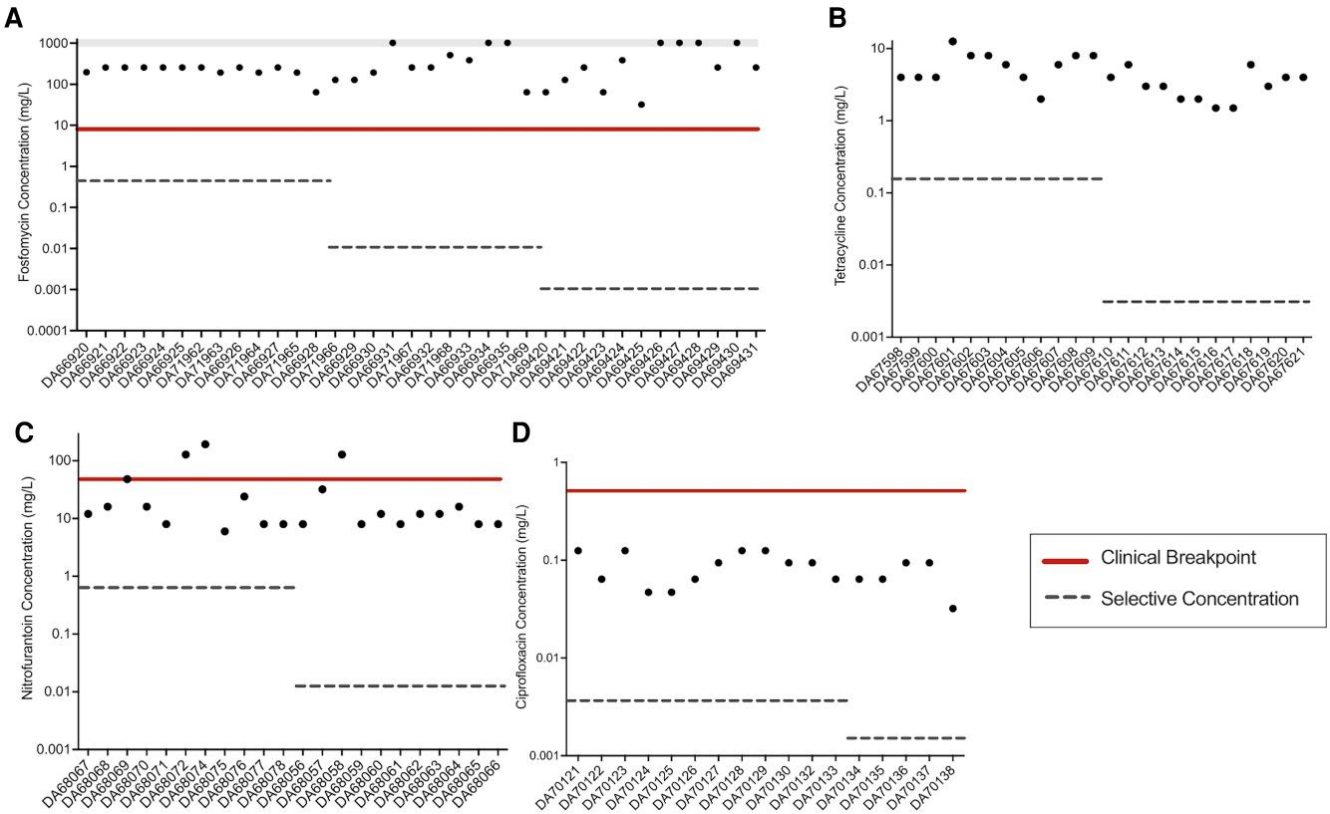
de novo-resistant mutants selected at subMICs of antibiotics have resistance levels that are above the clinical break-points. Resistance levels of mutants selected at subMICs of 1/4th, 1/200^th^, and 1/2000th MIC_susceptible_ are plotted for (*A*) fosfomycin, those selected at subMICs 1/4th and 1/200th MIC_susceptible_ are plotted for (*B*) tetracycline, (*C*) nitrofurantoin, and those selected at subMICs of 1/4th and 1/10th MIC_susceptible_ are plotted for (*D*) ciprofloxacin. The absolute antibiotic concentrations used for selection are shown as dotted lines, while the clinical breakpoints are shown as solid lines. For fosfomycin, data points shown in the gray bar represent MIC values that are greater than the maximum that could be measured using an E-test, that is, the MIC for these strains is >1024 mg/L.

Selection of resistant mutants occurs over a broad range of subMICs in our experiments, allowing us to determine the minimum selective concentration (MSC) for each of the four antibiotics used in our study (fig. 2, Table 1 and supplementary Table S2, Supplementary Material online). The lowest antibiotic concentration at which the selection of resistant mutants was observed is reported as the MSC in our dataset. To determine the MSC for resistant mutants with different levels of resistance, we screened for the emergence of mutants at antibiotic concentrations of 2 × -, 4 × -, 8 × -, and 16 × of MIC_susceptible._ As summarized in figure 2, depending on the antibiotic, the lowest concentration at which resistant mutants are selected (i.e., MSC) is either 1/20th (ciprofloxacin), 1/200th (tetracycline), or 1/2000th (fosfomycin and nitrofurantoin) of MIC_susceptible_. In addition, and as expected, the higher the antibiotic concentration used for selection, the higher the resistance level of the resistant mutants. This was observed for nitrofurantoin, tetracycline, and ciprofloxacin, but not for fosfomycin, where even the lowest concentration (1/2000th of MIC_susceptible_) was selected for mutants that could grow at all tested concentrations. Importantly, the subMIC exposure resulted in the selection of mutants with resistance levels above the clinical break-point (fosfomycin and nitrofurantoin) or just below (ciprofloxacin), whereas for tetracycline, no clinical breakpoint has been set for *E. coli* (fig. 3).

**Table 1.** Minimal Selective Concentrations (MSC) for Resistant Mutants With Different Levels of Resistance (2–64×MIC) on Fosfomycin, Tetracycline, Nitrofurantoin, and Ciprofloxacin.

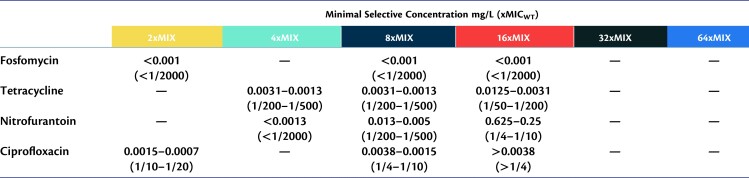

MSC values were calculated based on the enrichment of resistant mutants in evolution experiments at subMICs of antibiotics.

### Clinically Relevant Resistance Mutations Can be Selected at subMICs of Antibiotics

To identify the resistance mutations that were enriched in these experiments, we, whole-genome sequenced a subset of resistant clones isolated at the end-point of the evolution experiment for each antibiotic. Thus, eight clones were whole-genome sequenced from lineages each evolving at antibiotic concentrations of 1/4th and 1/200th of MIC_susceptible_. For fosfomycin, resistant clones selected at antibiotic concentrations of 1/2000th of MIC_susceptible_ were also whole-genome sequenced, while ciprofloxacin-resistant clones selected at 1/10th of MIC_susceptible_ (instead of 1/200th of MIC_susceptible_) were whole-genome sequenced. To ensure that we sequenced mutants with potentially different resistance mutations, we first determined the growth rate of the isolated mutants at different concentrations of the appropriate antibiotic. This allowed us to choose mutants with different growth rates (and potentially different types of mutations) for whole-genome sequencing (supplementary fig. S2, Supplementary Material online). We also determined if any of the selected mutants were mutator strains by performing the Luria–Delbruck fluctuation test with rifampicin resistance as the test marker. If the resistant clone was identified as a mutator strain then, wherever possible, it was not whole-genome sequenced because of the difficulty in making any meaningful genotype–phenotype correlations in mutants carrying many mutations.

**Fig. 4.**
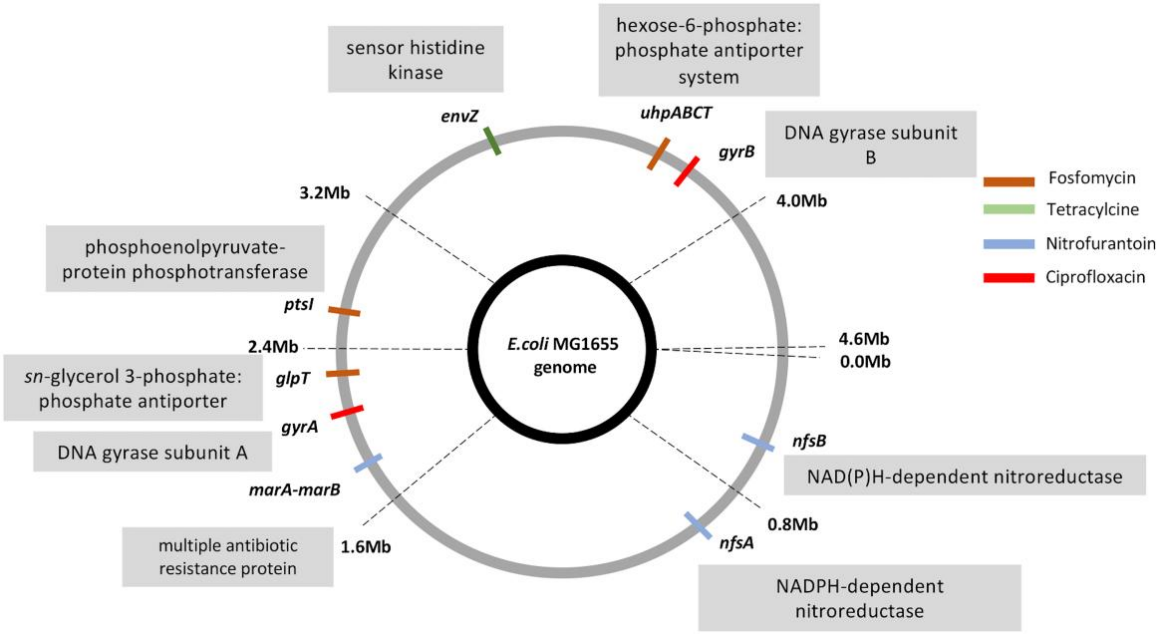
Whole-genome sequencing of resistant mutants shows the prevalence of clinically relevant resistance mutations selected at subMICs of antibiotics. Resistant mutants selected at the subMICs were whole-genome sequenced and clinically relevant resistance mutations are indicated. Different colors represent the selection of different antibiotics.

This analysis demonstrates the presence of multiple clinically relevant antibiotic resistance mutations among the isolated resistant clones (fig. 4, supplementary fig. S3 and Table S3, Supplementary Material online). For fosfomycin, this included mutations in genes *glpT* (glycerol 3-phosphate:phosphate antiporter), *uhpT* (hexose-6 phosphate:phosphate antiporter), *uhpC* (inner membrane protein sensing glucose 6-phosphate), and *uhpA* (transcriptional activator for genes encoding proteins sensing glucose 6-phosphate). Mutations in these genes have been shown to confer fosfomycin resistance by reducing fosfomycin uptake (Nilsson et al. 2003; Fu et al. 2015). Resistance to the antibiotic nitrofurantoin was linked to mutations in genes *nfsA* (nitroreductase), *nfsB* (nitroreductase), and a deletion in the intergenic region between the genes *marA-marB* (“multiple antibiotic resistance” genes). Proteins encoded by genes *nfsA* and *nfsB* are enzymes known to activate the inactive form of nitrofurantoin inside the cell, and mutations that result in the loss of function or reduced function in these genes would thus result in lower levels of the activated antibiotic inside the cell (Sandegren et al. 2008; Wan et al. 2021). Additionally, proteins encoded by the *marA-marB* genes are known to regulate the expression of genes involved in conferring antibiotic resistance and resistance to oxidative stress (Barbosa and Levy 2000; Chollet et al. 2002). Ciprofloxacin-resistant mutants included mutations in genes *gyrA* (DNA gyrase subunit A) and *gyrB* (DNA gyrase subunit B). These are the target genes for ciprofloxacin, and mutations in these genes result in reduced binding of ciprofloxacin to DNA gyrase (Lee et al. 2005; Garoff et al. 2020). Tetracycline-resistant mutants had mutations in the gene *envZ* (sensor histidine kinase). Although it is unclear how mutations in this gene confer resistance toward tetracycline, they have been shown to be clinically relevant for other antibiotics (Adler et al. 2016).

### Both Increased Resistance and Media Adaptation Contribute to the Increased Fitness at subMIC of Antibiotics

**Fig. 5.**
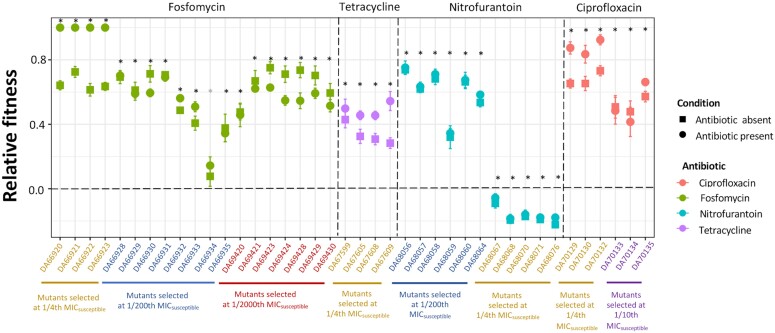
Relative fitness of resistant mutants in the presence and absence of the antibiotic. Forty resistant mutants isolated at the endpoint of the experiment competed with the susceptible ancestral strains in the presence (circles) and absence of the antibiotic (squares). The concentration of the antibiotic used in competition experiments was the same as that used during the selection experiments. Relative fitness is given as selection coefficients. Different colors represent different antibiotics. Eight independent competitions were performed in each case. The error bars represent a standard deviation. Two-tailed Student’s t-test was performed to test for statistically significant differences at *P* = 0.05. Black * indicates that relative fitness is statistically different from 0, both in the presence and absence of the antibiotic, while gray * indicates that relative fitness is statistically different from 0 only in the presence of the antibiotic.

Our experimental design allowed us to determine the contributions of resistance evolution and growth environment adaptation to the enrichment of mutants at the subMIC levels of antibiotics. To this end, we measured the relative fitness of 40 resistant mutants (isolated from the end-point of the evolution experiment) both in the presence and absence of the antibiotic by competition with the wild-type susceptible ancestral strain. Out of these 40 resistant mutants, 34 showed an increase in fitness both in the presence and absence of the antibiotic, one had higher fitness than the wild-type susceptible strain in the presence of the antibiotic but had the same fitness in its absence, and five showed a reduction in fitness under both these conditions (fig. 5). The latter observation is peculiar, but it most likely indicates the presence of non-transitive fitness interactions in our experiments (Paquin and Adams 1983; Rainey and Travisano 1998; Buskirk et al. 2020), such that a competition with the ancestral parent strain does not correctly reflect the fitness trajectory of the evolving population. These mutants were not investigated further.

**Fig. 6.**
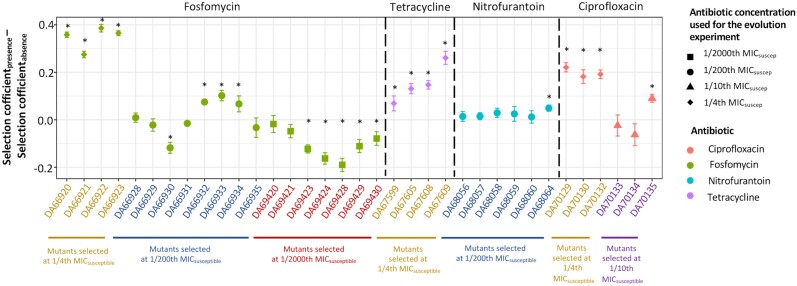
Determining the contribution of increased resistance to the increase in fitness. The contribution of increased resistance to an increase in fitness was determined by plotting the difference between the selection coefficients calculated from competition experiments between the resistant mutants and susceptible strains in the presence and absence of the antibiotic. A value higher than 0 denotes a measurable contribution of increased resistance to the increase in fitness. Values significantly different from 0 are marked with a * in the figure. The error bars represent standard deviations calculated based on standard deviations from individual means. Different shapes represent the antibiotic concentrations where selection for specific resistant mutants was observed and different colors represent different antibiotics. Two-tailed Student’s t-test was performed to test for statistically significant differences at *P* = 0.05.

To determine the contribution of increased antibiotic resistance to the increase in fitness among the remaining 35 resistant mutants, we calculated the difference between the selection coefficients measured in the presence and absence of the antibiotic (fig. 6). Out of the 35 resistant mutants investigated, 16 mutants showed a measurable and statistically significant effect of the presence of the antibiotic on fitness (fig. 6). Among the remaining 19 mutants, 13 did not have a value that was statistically different from 0. It is possible that in these 13 cases, the fitness advantage due to increased antibiotic resistance was too small to be measured by our competition assay. The difference between the selection coefficients for the remaining six mutants was statistically lower than 0, suggesting potentially complex interactions between mutations conferring resistance and those conferring adaptation to the growth environment. Despite this complexity, our data convincingly demonstrate that under these conditions, there is concomitant selection for increased resistance and adaptation to the growth environment.

**Fig. 7.**
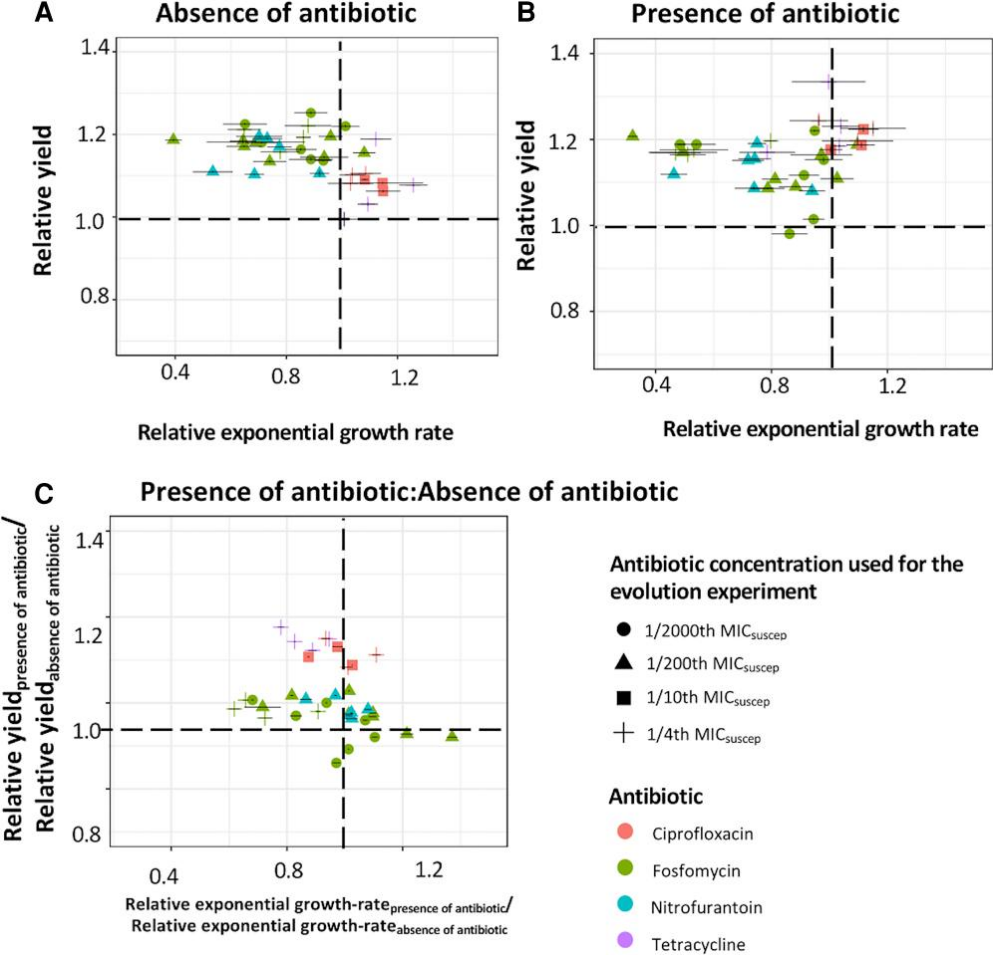
Contribution of the exponential growth rate and growth yield (stationary phase population density) to mutant fitness. Relative exponential growth rate and relative growth yield (with respect to the susceptible ancestral strain) were calculated for the resistant mutants in the (*A*) absence and (*B*) presence of the antibiotic. The wild-type values were set as 1 in each case and are shown as dotted lines. (*C*) The ratios between these measurements in the presence and absence of the antibiotic were calculated to determine the contribution of these fitness components to the increase in fitness. A value different from 1 would represent a measurable contribution of either an increased exponential growth rate or growth yield to the increased fitness. Error bars along both the x-axis and y-axis represent standard deviations. Different shapes represent antibiotic concentrations where selection for specific resistant mutants was observed, while different colors represent different antibiotics.

We also investigated the growth curves for each of these 35 resistant mutants to determine the relative contribution of an altered exponential growth rate and/or growth yield (population density in stationary phase) to increased fitness at subMICs of antibiotics (fig. 7*A* and *B*). A majority of the resistant mutants showed a higher relative growth yield both in the presence and absence of the antibiotic (when compared with the susceptible ancestral strain). For a small subset of mutants, we also observed a higher relative exponential growth rate for the resistant mutants both in the presence and absence of the antibiotic. Overall, these findings suggest that selection for increased yield was the predominant adaptive response during these serial passage conditions. We further investigated the correlation between increased resistance and these fitness components by comparing the relative exponential growth rate and growth yield for each mutant in the presence and absence of the antibiotic (fig. 7*C*). About half (18 out of 35) of the resistant mutants showed either an increased growth-yield (10 out of 35) or higher exponential growth rate (8 out of 35) that was due to the presence of the antibiotic, suggesting that selection for an increased growth yield and a higher exponential growth rate contributed equally toward resistance evolution at subMIC of antibiotics. Based on the present data, we cannot determine which type of mutants emerged first during selection.

**Fig. 8.**
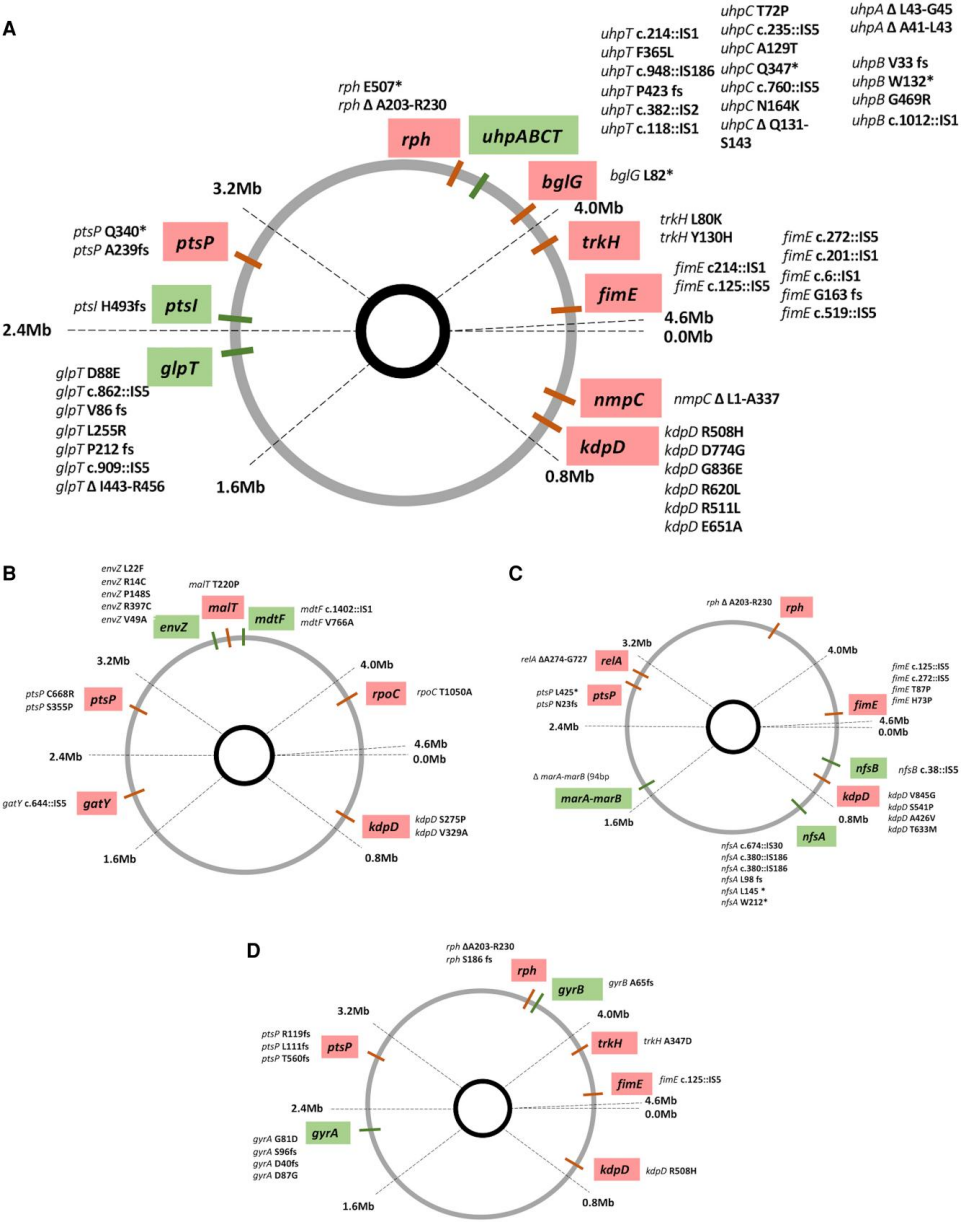
Unique mutations contribute to the increase in resistance and growth environment adaptation. Whole-genome sequencing of multiple clones identified 95 unique mutations across 24 genes that potentially contribute to either an increase in resistance (marked as green) or adaptation to the growth (non-antibiotic) environment (marked as red). These are individually shown for each antibiotic (*A*-fosfomycin, *B*-tetracycline, *C*-nitrofurantoin, and *D*-ciprofloxacin). The mutations observed in the different mutants are listed next to each gene.

Whole-genome sequencing of these resistant clones allowed us to identify the genetic changes that either contributed to the mutant becoming more resistant or better adapted to the growth environment. To determine the contribution of individual mutations, we first performed a literature search to identify mutations that are known to contribute to either resistance evolution or those that are known to result in adaptation to the growth environment (supplementary fig. S3 and supplementary Table S3, Supplementary Material online). In addition, we also reconstructed specific mutations to investigate them individually, including the mutations in genes *kdpD* (Arg511Lys), *glpT* (Asp88Glu), *nfsA* (Trp 212*), and *envZ* (Val49Ala). A total of 95 unique mutations were identified across 24 genes (fig. 8*A*–*D*). Based on the list of mutations identified from the literature and those that were reconstructed, 47 of them could potentially increase fitness by increasing resistance, while 48 of them could potentially confer adaptation to the growth environment (fig. 8*A*–*D*). Thus, at all the subMICs for the different antibiotics investigated, there was a selection for mutations conferring antibiotic resistance and growth environment adaptation.

### Resistant Mutants Display Cross-resistance to Other Antibiotics

To determine if resistant mutants selected in our experiments were cross-resistant to other antibiotics, we measured the MICs of several different antibiotics for 83 resistant clones isolated from the end-point of our experiment. Cross-resistance was observed for clones selected on tetracycline and nitrofurantoin, where the former showed cross-resistance to fosfomycin and ciprofloxacin, and the latter demonstrated cross-resistance to fosfomycin. (supplementary Table S4, Supplementary Material online). The tetracycline-resistant mutants did not have any known mutations that confer high-level of fosfomycin resistance, whereas the nitrofurantoin-resistant mutants demonstrating cross-resistance to fosfomycin had a mutation in the *uhpT* gene, which has previously been implicated in fosfomycin resistance (Nilsson et al. 2003).

## Discussion

### The Minimal Selective Concentration for the Enrichment of de novo Resistance Mutations is Antibiotic Dependent

One main objective of this study was to determine the MSC for de novo resistance mutations for several antibiotics. The choice of antibiotics was motivated by clinical relevance and included fosfomycin, nitrofurantoin, and ciprofloxacin, which are all used for *E. coli* infections. Furthermore, clinically relevant resistance mutations are well characterized for these antibiotics, and in most cases, are a result of de novo occurring mutations. We also included tetracycline in our study because of its human and veterinary use (for other infections than those caused by *E. coli*) and common occurrence as an anthropogenic pollutant in several different environments. These four antibiotics also inhibit the growth of the bacterium in different ways by interacting with a diverse array of cellular processes. Thus, fosfomycin interacts with the MurA protein (Marquardt et al. 1994; Silver 2017) which is involved in peptidoglycan synthesis (cell-wall synthesis), ciprofloxacin interacts with the DNA gyrase enzyme (Barnard and Maxwell 2001; Mustaev et al. 2014) (DNA replication), tetracycline interacts with the ribosome (Chopra and Roberts 2001) (protein synthesis), while nitrofurantoin is a pro-dug that is activated to a free radical compound, which is suggested to have multiple targets within the cell (Jenkins and Bennett 1976; McOsker and Fitzpatrick 1994). Despite these different modes of actions, selection for antibiotic resistance was observed for all four antibiotics, but the MSC varied between the different antibiotics, both with regard to absolute concentration and relative to the MIC_susceptible_. Thus, in our experiments, we observed selection for fosfomycin and nitrofurantoin resistance mutations at antibiotic concentrations of 1/2000th of MIC_susceptible_, for tetracycline at 1/500th of MIC_susceptible_, and for ciprofloxacin-resistant mutants, selection was observed at an antibiotic concentration of 1/20th of MIC_susceptible_. Although this difference in MSCs for different antibiotics is in accordance with previously published studies (Sanz-García et al. 2022), the selection of clinically relevant resistance mutations for fosfomycin at 1/2000th of MIC_susceptible_ is 10 times lower than the previously published values for MSCs for any antibiotic, accentuating the role of very low antibiotic concentrations as a selective force that enriches antibiotic resistance mutations in the environment.

### Pervasive Selection of Clinically Relevant Mutations at subMICs of Antibiotics

Another important observation in this study was the pervasive selection of clinically relevant antibiotic-resistance mutations. Thus, mutations in the genes *glpT* and genes of the *uhp* operon were enriched under the subMICs of fosfomycin, mutations in genes *nfsA* and *nfsB* were selected under the subMICs of nitrofurantoin, mutations in genes *gyrA* and *gyrB* were selected under the subMICs of ciprofloxacin, and mutations in genes *envZ* were selected under the subMICs of tetracycline. Mutations in all these genes have previously been identified in clinical isolates with resistance to the respective antibiotics (Lee et al. 2005; Fu et al. 2015; Adler et al. 2016; Wan et al. 2021), except for the mutations in gene *envZ* which has been shown to be clinically relevant for other antibiotics (Adler et al. 2016). Thus, not only does this study demonstrate the selection of resistance mutations at extremely low concentrations, but also shows the selection of known clinically relevant mutations at these concentrations. Whole-genome sequencing analysis of multiple resistant mutants for each selective regime allowed us to determine that 47 unique mutations were observed across the above-mentioned targets (fig. 8*A*–*D*), further highlighting the pervasive selection for antibiotic resistance evolution at subMICs of antibiotics. This observation of a large number of mutations for increased resistance in clinically relevant target genes is worrisome, since similar antibiotic concentrations have been shown to be present in many different environments (Chee-Sanford et al. 2009; Heuer et al. 2011; McManus 2014; Gao et al. 2015). This suggests that several types of environments could serve as points of emergence and spread of these clinically relevant mutations. Furthermore, some of these mutations also conferred cross-resistance to other antibiotics than those they were selected on, making them especially problematic.

### Adaptation to the Growth Environment Plays a Significant Role Under the subMIC Selection Regime

Another significant observation in our study was that besides the selection for increased resistance, adaptation to the growth environment (non-antibiotic related) was also a significant selective pressure. Thus, out of the 40 resistant mutants isolated from populations evolving at the subMICs of different antibiotics and where fitness was measured using competition experiments, 34 had higher fitness than the starting susceptible ancestral strain, even in the absence of the antibiotic. Whole-genome sequencing analysis of these resistant clones further shows that a total of 48 mutations could potentially increase fitness to the growth environment across these isolated resistant clones (fig. 8*A*–*D*). Intuitively, evolution under the subMIC selective regime could either proceed via the evolution of “specialist” types where adaptation against the antibiotics could represent one fitness peak, while adaptation to the growth environment might represent a second fitness peak. Alternatively, these environments could select for “generalist” strains that are adapted to both the environment and against the antibiotics, implying the occurrence of multiple selective sweeps, where the adaptive mutations for both these selective pressures occur in the same background, and/or that pleiotropic mutations that confer both resistance and media adaptation are selected (Knöppel et al. 2017; Lamrabet et al. 2019). Our results suggest that the evolutionary trajectory under the subMIC selective regime is inclined toward the selection of these generalist strains that are both resistant to the antibiotics and better adapted to the growth environment. Although several studies have shown the evolution of antibiotic resistance at low antibiotic concentrations, the occurrence of adaptation to the growth environment in such environments is usually unaccounted for. Our results imply that the selection of resistance mutations under the subMIC selection regime should be extended to include mutations that confer a general growth adaptation, and their role in influencing the evolutionary outcome when the antibiotic selection is weak, should further be investigated.

## Materials and Methods

### Bacterial Strains, Antibiotics and Growth Conditions

The bacterial strain *Escherichia coli K-12* MG1655 was used as the susceptible parental strain for all the experiments. All the evolved resistant clones that were investigated in this study are listed in supplementary Table S1, Supplementary Material online. The antibiotics used for the evolution experiments included fosfomycin (MIC_susceptible/Etest_ = 0.380 mg/L and MIC_susceptible/broth micro._= 2 mg/L), tetracycline (MIC_susceptible/Etest_ = 0.750 mg/L and MIC_susceptible/broth micro._= 0.625 mg/L), nitrofuranotoin (MIC_susceptible/Etest_ = 2 mg/L and MIC_susceptible/broth micro._= 2.5 mg/L), and ciprofloxacin (MIC_susceptible/Etest_ = 0.012 mg/L and MIC_susceptible/broth micro._ = 0.015 mg/L). The liquid and solid media used for all the experiments were Mueller–Hinton broth and Mueller–Hinton Agar (Becton Dickinson, MD, USA). All the experiments were performed at 37 °C. Glucose-6-phosphate was added to the media at a concentration of 50 mg/L for experiments involving fosfomycin.

### Experimental Evolution Set-up and Determining the Emergence and Frequency of Resistant Mutants

The evolution experiments are performed in 96-well plates with eight lineages evolving per subMIC of the antibiotic (fig. 1). Eight replicate populations were also evolved in an antibiotic-free media and these served as control populations to determine the basal mutation rate for antibiotic resistance mutations. Several subMICs were used for each antibiotic, ranging from 1/4th of MIC_susceptible_ to 1/2000th of MIC_susceptible_. Each well contained 200 μl of the appropriate media, and the bacteria was allowed to grow for 12 h after which 20 μl of grown cells was transferred to 180 μl of fresh media. This resulted in ∼3.3 generations of growth per cycle. At different time points during the course of the experiment (supplementary fig. S1, Supplementary Material online), 1 μl from each well was plated on antibiotic-containing plates to determine the occurrence and frequency of resistant mutants. Since we were broadly interested in the evolutionary dynamics at each subMIC of the antibiotic and not in the individual population-level dynamics, we mixed 1 μl each from four lineages that were evolving under the same condition and plated the mixture on a single antibiotic-containing plate. Thus, the frequency measured is a cumulative frequency of resistant mutants combined from four lineages. In each case, dilutions of this mixture were also plated on non-antibiotic-containing plates to approximate the population size for the lineages. MSC is reported as the lowest antibiotic concentration where enrichment of the resistant mutants was observed. Fixation of high-level resistant mutants was assumed when a bacterial lawn was observed on the antibiotic-containing plates. The antibiotic concentrations used to screen for resistant mutants ranged from 2×-MIC_susceptible_ to 16×-MIC_susceptible_.

### Growth Analysis of Resistant Mutants and Identification of Mutator Strains

Growth curves for all the resistant mutants were plotted by measuring growth using a BioscreenC analyzer at OD_600_, with measurements taken every 4 min. A 1:1,000 dilution of an overnight-grown culture was used to start the experiment, and the maximum exponential growth rate was calculated using the optical density (OD_600_) values between 0.02 and 0.09. Both the susceptible and resistant strains were grown in the absence and presence of different concentrations of the antibiotic to determine phenotypic differences between the resistant mutants. Six replicates were used in each case.

The potential occurrence of hyper-mutators among the isolated resistant mutants was determined by calculating the resistant mutant frequency for the antibiotic rifampicin. Briefly, 100 μl from an overnight-grown culture (for both resistant and susceptible strains) was plated on plates containing 100 μg/ml of rifampicin, and the plates were then incubated at 37 °C for 20 h. Four biological replicates were grown for each strain. In each case, dilutions were also plated on non-antibiotic-containing plates to determine the total number of cells being plated so as to calculate the frequency of rifampicin-resistant mutants.

### Whole-genome Sequencing and Identification of Mutations

To identify resistance mutations and other adaptive mutations in the evolving populations, single clones were isolated and whole-genome sequenced. DNA extraction was done with 1 ml of overnight-grown cultures using the Epicenter DNA extraction kit following the manufacturer’s protocol. Illumina’s Nextera XT kit was used to make whole-genome DNA libraries (2 × 300) that were then sequenced on Illumina’s Miseq platform. Samples were dual-indexed and pooled together. The average whole-genome coverage per sample was 30 × . Analysis of the fastq files obtained from Miseq sequencing was performed using CLC genomics Workbench version 11. This tool was used to identify point mutations and small indels. To identify large deletions or transposon movements, the fastq files were also analyzed using Breseq (version 0.27.1a) (Deatherage and Barrick 2014). In each case, the reads were mapped onto the susceptible *E.coli* K-12 MG1655 strain that was also whole-genome sequenced and was the ancestral strain for all the evolved resistant mutants.

### Determination of the Minimum Inhibitory Concentration

The MIC for different antibiotics for all the strains used in this study was determined using Etests, as per the instructions from the manufacturer (bioMérieux, Marcy l'Étoile, France). Briefly, overnight-grown cultures were diluted 1:20 in phosphate-buffered saline and then spread on Mueller–Hinton plates using a cotton swab. The Etests were then placed on the plates after which these were incubated at 37 °C for 16–18 h. Two biological replicates were done in each case.

### Determining the Relative Fitness of Resistant Mutants by Competition Experiments

Competition experiments were performed by genetically tagging the different strains with genes encoding for fluorescent markers [yellow fluorescent protein (YFP) and blue fluorescent protein (BFP)]. Fluorescently labeled strains were grown overnight in Mueller–Hinton media in the absence of antibiotics, and then mixed in a 1:1 ratio to start the competition experiments. All competitions were performed in 96-well plates, where 20 μl of an overnight-grown culture was transferred to 180 μl of media each day, resulting in ∼3.3 generations per day. The frequencies for each strain were determined at the beginning of the experiment and after ∼10 generations. Strain frequencies at generations ∼20 and ∼30 were also measured for competition experiments involving nitrofurantoin-resistant mutants. Measurement of fluorescently tagged cells was done by making 40-fold dilutions of cultures from each well in phosphate-buffered saline. One Hundred Thousand cells were counted and the fraction of BFP and YFP positive cells was determined by flow cytometry (MACSQuant VYB, Miltenyi Biotec). Competition experiments for each strain were also performed by swapping the fluorescent markers so as to determine any fitness costs that might have been associated with the expression of the fluorescent proteins. The rate of change of the different strains is plotted as the natural log of the ratio of different strains at different timepoints. The slope of this line is calculated and reported as the selection coefficient. Eight replicates were used in each case. A two-tailed Student’s t-test was performed to test for statistically significant differences at *P* = 0.05.

### Re-constructing Resistance-conferring Mutations Using λ-red Recombineering

A subset of the different resistance mutations was reconstructed using the λ-red recombineering technique (Yu et al. 2000). This involved using a 500 μl of an overnight-grown culture of *E. coli* K-12 MG1655 strain containing the pSIM5-Tet plasmid (Koskiniemi et al. 2011) (grown at 30 °C) and inoculating 50 ml of LB broth containing 10 mg/L of tetracycline. These cells were allowed to grow at 30 °C until the optical density (OD_600_) reached ∼0.2, after which they were transferred to a 42 °C shaking water bath for 20 min. At the end of 20 min, the cells were placed on ice for 5 min and then washed thrice with 10% glycerol. The pellet was resuspended in the residual 10% glycerol. A *cat-sacB* marker was then inserted in these cells at the desired location by electroporation. The *cat-sacB* marker, which was generated by PCR-amplification and consisted of over-hangs that were homologous to the site of insertion on the *E. coli* genome, consists of the selectable *cat* marker (allowing selection on chloramphenicol plates) and the counter-selectable *sacB* marker (resulting in cell-death in sucrose-containing plates). Once the marker is inserted at the desired position, similar steps are followed as above, to replace it with a single-stranded oligonucleotide containing the desired mutation and counter selecting them on plates containing 5% sucrose. Clones that can grow on sucrose-containing plates were isolated and the presence of the desired mutation was confirmed by Sanger sequencing.

## Supplementary Material

msad010_Supplementary_Data

## Data Availability

The data underlying this article are available in the article and its online supplementary material. Fastq files with whole-genome sequences of resistant mutants have been uploaded at NCBIs SRA database with the bioproject accession number PRJNA906735.

## References

[msad010-B1] Adler M , Anjum M, Andersson DI, Sandegren L. 2016. Combinations of mutations in envZ, ftsI, mrdA, acrB and acrR can cause high-level carbapenem resistance in Escherichia coli. J Antimicrob Chemother. 71(5):1188–1198. 26869688 10.1093/jac/dkv475

[msad010-B2] Baquero F , Negri MC. 1997. Selective compartments for resistant microorganisms in antibiotic gradients. BioEssays News Rev Mol Cell Dev Biol. 19(8):731–736. 10.1002/bies.950190814 9264256

[msad010-B3] Barbosa TM , Levy SB. 2000. Differential expression of over 60 chromosomal genes in *Escherichia coli* by constitutive expression of MarA. J Bacteriol. 182(12):3467–3474. 10852879 10.1128/jb.182.12.3467-3474.2000 PMC101932

[msad010-B4] Barnard FM , Maxwell A. 2001. Interaction between DNA gyrase and quinolones: effects of alanine mutations at GyrA subunit residues Ser(83) and Asp(87). Antimicrob Agents Chemother. 45(7):1994–2000. 11408214 10.1128/AAC.45.7.1994-2000.2001 PMC90591

[msad010-B5] Berge ACB , Atwill ER, Sischo WM. 2005. Animal and farm influences on the dynamics of antibiotic resistance in faecal *Escherichia coli* in young dairy calves. Prev Vet Med. 69(1–2):25–38. 15899294 10.1016/j.prevetmed.2005.01.013

[msad010-B6] Björkman J , Nagaev I, Berg OG, Hughes D, Andersson DI. 2000. Effects of environment on compensatory mutations to ameliorate costs of antibiotic resistance. Science. 287(5457):1479–1482. 10688795 10.1126/science.287.5457.1479

[msad010-B7] Buskirk SW , Rokes AB, Lang GI. 2020. Adaptive evolution of nontransitive fitness in yeast. Elife. 9:e62238. 10.7554/eLife.62238 PMC7886323 33372653

[msad010-B8] Chee-Sanford JC , Mackie RI, Koike S, Krapac IG, Lin Y-F, Yannarell AC, Maxwell S, Aminov RI. 2009. Fate and transport of antibiotic residues and antibiotic resistance genes following land application of manure waste. J Environ Qual. 38(3):1086–1108. 19398507 10.2134/jeq2008.0128

[msad010-B9] Chollet R , Bollet C, Chevalier J, Malléa M, Pagès J-M, Davin-Regli A. 2002. mar Operon involved in multidrug resistance of Enterobacter aerogenes. Antimicrob Agents Chemother. 46(4):1093–1097. 11897595 10.1128/AAC.46.4.1093-1097.2002 PMC127096

[msad010-B10] Chopra I , Roberts M. 2001. Tetracycline antibiotics: mode of action, applications, molecular biology, and epidemiology of bacterial resistance. Microbiol Mol Biol Rev MMBR. 65(2):232–260. 11381101 10.1128/MMBR.65.2.232-260.2001 PMC99026

[msad010-B11] Davies J , Spiegelman GB, Yim G. 2006. The world of subinhibitory antibiotic concentrations. Curr Opin Microbiol. 9(5):445–453. 16942902 10.1016/j.mib.2006.08.006

[msad010-B12] Deatherage DE , Barrick JE. 2014. Identification of mutations in laboratory-evolved microbes from next-generation sequencing data using breseq. Methods Mol Biol. 1151:165–188. 24838886 10.1007/978-1-4939-0554-6_12 PMC4239701

[msad010-B13] de Sousa JM , Sousa A, Bourgard C, Gordo I. 2015. Potential for adaptation overrides cost of resistance. Future Microbiol. 10(9):1415–1431. 26343510 10.2217/fmb.15.61 PMC4663674

[msad010-B14] Drekonja DM , Trautner B, Amundson C, Kuskowski M, Johnson JR. 2021. Effect of 7 vs 14 days of antibiotic therapy on resolution of symptoms among afebrile men with urinary tract infection: a Randomized Clinical Trial. JAMA. 326(4):324–331. 34313686 10.1001/jama.2021.9899 PMC8317010

[msad010-B15] Fu Z , Ma Y, Chen C, Guo Y, Hu F, Liu Y, Xu X, Wang M. 2015. Prevalence of fosfomycin resistance and mutations in murA, glpT, and uhpT in methicillin-resistant Staphylococcus aureus strains isolated from blood and cerebrospinal fluid samples. Front Microbiol. 6:1544. 26793179 10.3389/fmicb.2015.01544 PMC4707275

[msad010-B16] Gao L , Hu J, Zhang X, Wei L, Li S, Miao Z, Chai T. 2015. Application of swine manure on agricultural fields contributes to extended-spectrum β-lactamase-producing *Escherichia coli* spread in Tai’an, China. Front Microbiol. 6:313. 25926828 10.3389/fmicb.2015.00313 PMC4396445

[msad010-B17] Garoff L , Pietsch F, Huseby DL, Lilja T, Brandis G, Hughes D. 2020. Population bottlenecks strongly influence the evolutionary trajectory to fluoroquinolone resistance in *Escherichia coli*. Mol Biol Evol. 37(6):1637–1646. 32031639 10.1093/molbev/msaa032 PMC7253196

[msad010-B18] Gifford DR , Moss E, MacLean RC. 2016. Environmental variation alters the fitness effects of rifampicin resistance mutations in *Pseudomonas aeruginosa*. Evol Int J Org Evol. 70(3):725–730. 10.1111/evo.12880 26880677

[msad010-B19] Gu Y , Huang L, Wu C, Huang J, Hao H, Yuan Z, Cheng G. 2021. The evolution of fluoroquinolone resistance in Salmonella under exposure to sub-inhibitory concentration of enrofloxacin. Int J Mol Sci. 22(22):12218. 34830098 10.3390/ijms222212218 PMC8619427

[msad010-B20] Gullberg E , Albrecht LM, Karlsson C, Sandegren L, Andersson DI. 2014. Selection of a multidrug resistance plasmid by sublethal levels of antibiotics and heavy metals. mBio. 5(5):e01918–e01914. 25293762 10.1128/mBio.01918-14 PMC4196238

[msad010-B21] Gullberg E , Cao S, Berg OG, Ilbäck C, Sandegren L, Hughes D, Andersson DI. 2011. Selection of resistant bacteria at very low antibiotic concentrations. PLoS Pathog. 7(7):e1002158. 10.1371/journal.ppat.1002158 PMC3141051 21811410

[msad010-B22] Heuer H , Schmitt H, Smalla K. 2011. Antibiotic resistance gene spread due to manure application on agricultural fields. Curr Opin Microbiol. 14(3):236–243. 21546307 10.1016/j.mib.2011.04.009

[msad010-B23] Huttner A , Kowalczyk A, Turjeman A, Babich T, Brossier C, Eliakim-Raz N, Kosiek K, de Tejada B M, Roux X, Shiber S, et al 2018. Effect of 5-day nitrofurantoin vs single-dose fosfomycin on clinical resolution of uncomplicated lower urinary tract infection in women: a randomized clinical trial. JAMA. 319(17):1781–1789. 29710295 10.1001/jama.2018.3627 PMC6134435

[msad010-B24] Huttner A , Verhaegh EM, Harbarth S, Muller AE, Theuretzbacher U, Mouton JW. 2015. Nitrofurantoin revisited: a systematic review and meta-analysis of controlled trials. J Antimicrob Chemother. 70(9):2456–2464. 26066581 10.1093/jac/dkv147

[msad010-B25] Jenkins ST , Bennett PM. 1976. Effect of mutations in deoxyribonucleic acid repair pathways on the sensitivity of *Escherichia coli* K-12 strains to nitrofurantoin. J Bacteriol. 125(3):1214–1216. 767322 10.1128/jb.125.3.1214-1216.1976 PMC236203

[msad010-B26] Knöppel A , Näsvall J, Andersson DI. 2017. Evolution of antibiotic resistance without antibiotic exposure. Antimicrob Agents Chemother. 61(11):e01495-17. 10.1128/AAC.01495-17 PMC5655081 28893783

[msad010-B27] Koskiniemi S , Pränting M, Gullberg E, Näsvall J, Andersson DI. 2011. Activation of cryptic aminoglycoside resistance in Salmonella enterica. Mol Microbiol. 80(6):1464–1478. 21507083 10.1111/j.1365-2958.2011.07657.x

[msad010-B28] Kot B . 2019. Antibiotic resistance among uropathogenic *Escherichia coli*. Pol J Microbiol. 68(4):403–415. 31880885 10.33073/pjm-2019-048 PMC7260639

[msad010-B29] Kümmerer K , Henninger A. 2003. Promoting resistance by the emission of antibiotics from hospitals and households into effluent. Clin Microbiol Infect. 9(12):1203–1214. 14686985 10.1111/j.1469-0691.2003.00739.x

[msad010-B30] Lamrabet O , Martin M, Lenski RE, Schneider D. 2019. Changes in intrinsic antibiotic susceptibility during a long-term evolution experiment with *Escherichia coli*. mBio. 10(2):e00189-19. 10.1128/mBio.00189-19 PMC6401480 30837336

[msad010-B31] Lee JK , Lee YS, Park YK, Kim BS. 2005. Mutations in the gyrA and parC genes in ciprofloxacin-resistant clinical isolates of Acinetobacter baumannii in Korea. Microbiol Immunol. 49(7):647–653. 16034208 10.1111/j.1348-0421.2005.tb03643.x

[msad010-B32] Leuin AS , Hartmann F, Viviano K. 2021. Administration of nitrofurantoin in dogs with lower urinary tract infections: 14 cases (2013–2019). J Small Anim Pract. 62(1):42–48. 33107048 10.1111/jsap.13252

[msad010-B33] Liu H , Yang Y, Sun H, Zhao L, Liu Y. 2018. Fate of tetracycline in enhanced biological nutrient removal process. Chemosphere. 193:998–1003. 29874776 10.1016/j.chemosphere.2017.11.136

[msad010-B34] Marquardt JL , Brown ED, Lane WS, Haley TM, Ichikawa Y, Wong CH, Walsh CT. 1994. Kinetics, stoichiometry, and identification of the reactive thiolate in the inactivation of UDP-GlcNAc enolpyruvoyl transferase by the antibiotic fosfomycin. Biochemistry. 33(35):10646–10651. 8075065 10.1021/bi00201a011

[msad010-B35] McManus PS . 2014. Does a drop in the bucket make a splash? Assessing the impact of antibiotic use on plants. Curr Opin Microbiol. 19:76–82. 25006016 10.1016/j.mib.2014.05.013

[msad010-B36] McOsker CC , Fitzpatrick PM. 1994. Nitrofurantoin: mechanism of action and implications for resistance development in common uropathogens. J Antimicrob Chemother. 33(Suppl A):23–30. 7928834 10.1093/jac/33.suppl_a.23

[msad010-B37] Mustaev A , Malik M, Zhao X, Kurepina N, Luan G, Oppegard LM, Hiasa H, Marks KR, Kerns RJ, Berger JM, et al 2014. Fluoroquinolone-gyrase-DNA complexes: two modes of drug binding. J Biol Chem. 289(18):12300–12312. 24497635 10.1074/jbc.M113.529164 PMC4007428

[msad010-B38] Nilsson AI , Berg OG, Aspevall O, Kahlmeter G, Andersson DI. 2003. Biological costs and mechanisms of fosfomycin resistance in *Escherichia coli*. Antimicrob Agents Chemother. 47(9):2850–2858. 12936984 10.1128/AAC.47.9.2850-2858.2003 PMC182645

[msad010-B39] Olofsson SK , Marcusson LL, Strömbäck A, Hughes D, Cars O. 2007. Dose-related selection of fluoroquinolone-resistant *Escherichia coli*. J Antimicrob Chemother. 60(4):795–801. 17635875 10.1093/jac/dkm265

[msad010-B40] Papich MG . 2017. Ciprofloxacin pharmacokinetics in clinical canine patients. J Vet Intern Med. 31(5):1508–1513. 28771831 10.1111/jvim.14788 PMC5598882

[msad010-B41] Paquin CE , Adams J. 1983. Relative fitness can decrease in evolving asexual populations of S. cerevisiae. Nature. 306(5941):368–370. 16752492 10.1038/306368a0

[msad010-B42] Pérez DS , Tapia MO, Soraci AL. 2014. Fosfomycin: uses and potentialities in veterinary medicine. Open Vet J. 4(1):26–43. 26623336 PMC4629597

[msad010-B43] Rainey PB , Travisano M. 1998. Adaptive radiation in a heterogeneous environment. Nature. 394(6688):69–72. 9665128 10.1038/27900

[msad010-B44] Roberts MC . 2003. Tetracycline therapy: update. Clin Infect Dis. 36(4):462–467. 12567304 10.1086/367622

[msad010-B45] Sandegren L , Lindqvist A, Kahlmeter G, Andersson DI. 2008. Nitrofurantoin resistance mechanism and fitness cost in *Escherichia coli*. J Antimicrob Chemother. 62(3):495–503. 18544599 10.1093/jac/dkn222

[msad010-B46] Sanz-García F , Hernando-Amado S, Martínez JL. 2022. Evolution under low antibiotic concentrations: a risk for the selection of Pseudomonas aeruginosa multidrug-resistant mutants in nature. Environ Microbiol. 24(3):1279–1293. 34666420 10.1111/1462-2920.15806

[msad010-B47] Sanz-García F , Sánchez MB, Hernando-Amado S, Martínez JL. 2020. Evolutionary landscapes of *Pseudomonas aeruginosa* towards ribosome-targeting antibiotic resistance depend on selection strength. Int J Antimicrob Agents. 55(6):105965. 10.1016/j.ijantimicag.2020.105965 32325206

[msad010-B48] Shenje J , Ifeoma Adimora-Nweke F, Ross IL, Ntsekhe M, Wiesner L, Deffur A, McIlleron HM, Pasipanodya J, Gumbo T, Mayosi BM. 2015. Poor penetration of antibiotics into pericardium in pericardial tuberculosis. EBioMedicine. 2(11):1640–1649. 26870790 10.1016/j.ebiom.2015.09.025 PMC4740291

[msad010-B49] Silver LL . 2017. Fosfomycin: mechanism and resistance. Cold Spring Harb Perspect Med. 7(2):a025262. 10.1101/cshperspect.a025262 PMC5287057 28062557

[msad010-B50] Song C , Zhang K-X, Wang X-J, Zhao S, Wang S-G. 2021. Effects of natural organic matter on the photolysis of tetracycline in aquatic environment: kinetics and mechanism. Chemosphere. 263:128338. 10.1016/j.chemosphere.2020.128338 33297264

[msad010-B51] Stanton IC , Murray AK, Zhang L, Snape J, Gaze WH. 2020. Evolution of antibiotic resistance at low antibiotic concentrations including selection below the minimal selective concentration. Commun Biol. 3(1):467. 32884065 10.1038/s42003-020-01176-w PMC7471295

[msad010-B52] Thabit AK , Fatani DF, Bamakhrama MS, Barnawi OA, Basudan LO, Alhejaili SF. 2019. Antibiotic penetration into bone and joints: an updated review. Int J Infect Dis. 81:128–136. 30772469 10.1016/j.ijid.2019.02.005

[msad010-B53] Wagenlehner FME , Vahlensieck W, Bauer HW, Weidner W, Piechota HJ, Naber KG. 2013. Prevention of recurrent urinary tract infections. Minerva Urol Nefrol. 65(1):9–20. 23538307

[msad010-B54] Wan Y , Mills E, Leung RCY, Vieira A, Zhi X, Croucher NJ, Woodford N, Jauneikaite E, Ellington MJ, Sriskandan S. 2021. Alterations in chromosomal genes nfsA, nfsB, and ribE are associated with nitrofurantoin resistance in *Escherichia coli* from the United Kingdom. Microb Genomics. 7:12. 10.1099/mgen.0.000702 PMC8767348 34860151

[msad010-B55] Wang S , Ji B, Zhang M, Gu J, Ma Y, Liu Y. 2021. Tetracycline-induced decoupling of symbiosis in microalgal-bacterial granular sludge. Environ Res. 197:111095. 10.1016/j.envres.2021.111095 33811864

[msad010-B56] Westhoff S , van Leeuwe TM, Qachach O, Zhang Z, van Wezel GP, Rozen DE. 2017. The evolution of no-cost resistance at sub-MIC concentrations of streptomycin in Streptomyces coelicolor. ISME J. 11(5):1168–1178. 28094796 10.1038/ismej.2016.194 PMC5437928

[msad010-B57] Wistrand-Yuen E , Knopp M, Hjort K, Koskiniemi S, Berg OG, Andersson DI. 2018. Evolution of high-level resistance during low-level antibiotic exposure. Nat Commun. 9(1):1599. 29686259 10.1038/s41467-018-04059-1 PMC5913237

[msad010-B58] Yu D , Ellis HM, Lee EC, Jenkins NA, Copeland NG, Court DL. 2000. An efficient recombination system for chromosome engineering in *Escherichia coli*. Proc Natl Acad Sci U S A. 97(11):5978. 10811905 10.1073/pnas.100127597 PMC18544

