# Supplementary material for: Pervasive Selection for Clinically Relevant Resistance and Media Adaptive Mutations at Very Low Antibiotic Concentrations
Source: Mol Biol Evol. 2023 Jan 11;40(1):msad010. doi: 10.1093/molbev/msad010 (PMC9887637; doi:10.1093/molbev/msad010)
Supplement: msad010_Supplementary_Data [file msad010_supplementary_data.pdf]

**Supplementary Fig. 1**. Increase in frequency of resistant mutants with different levels of resistance in populations evolving at sub-MICs of antibiotics. A) Fosfomycin B) Tetracycline C) Nitrofurantoin and D) Ciprofloxacin. Different levels of resistance was determined by plating the evolving populations at different antibiotic concentrations, which are shown in different colors in the figures

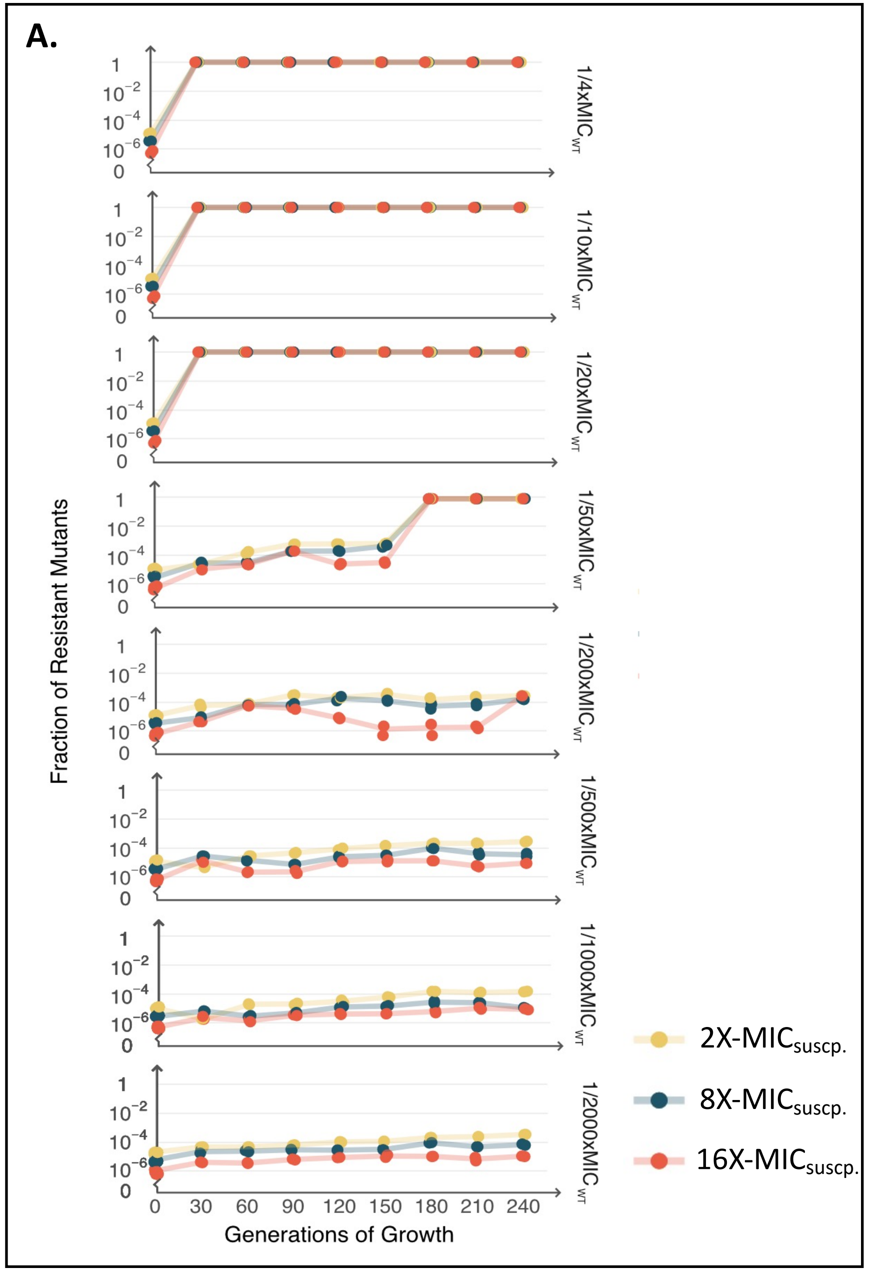


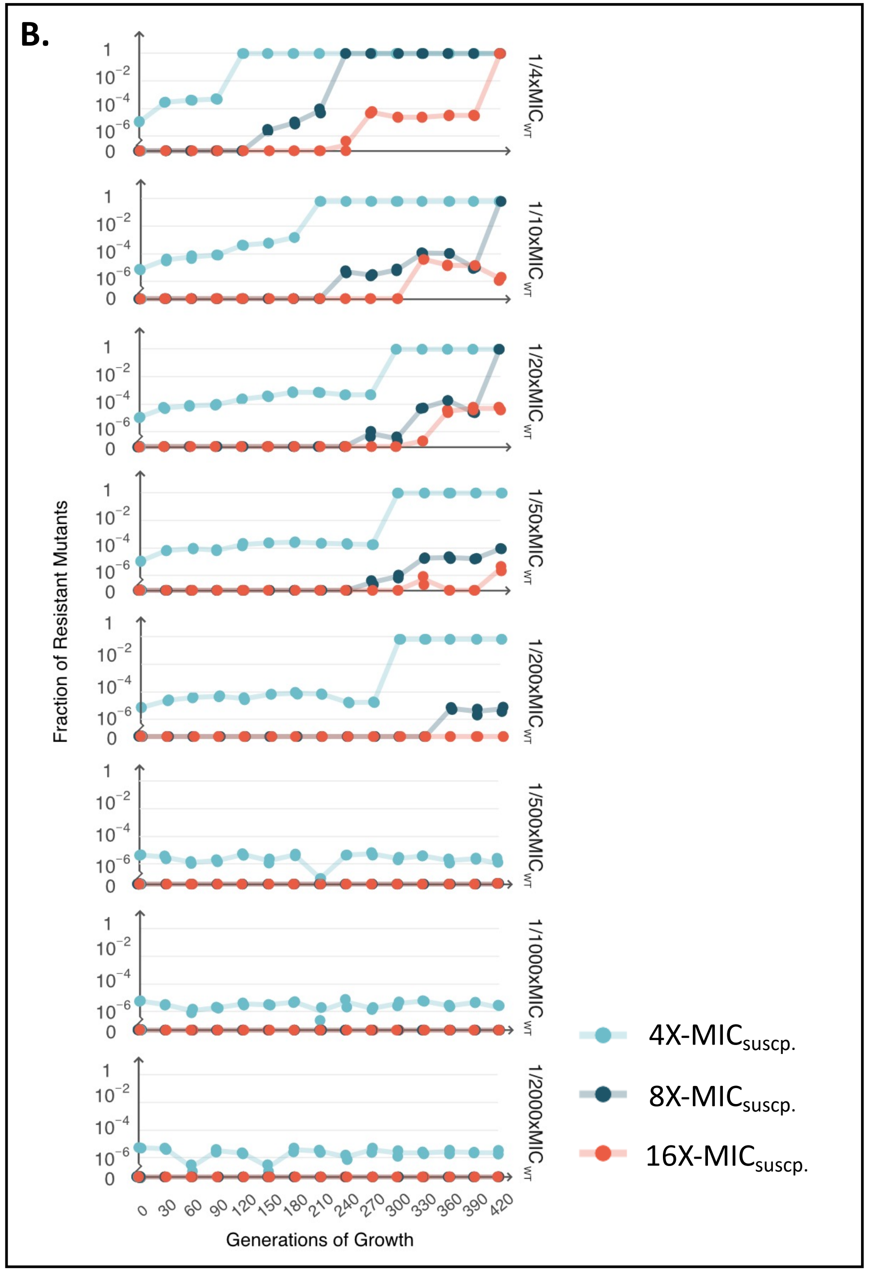

**Supplementary Fig. 1.**

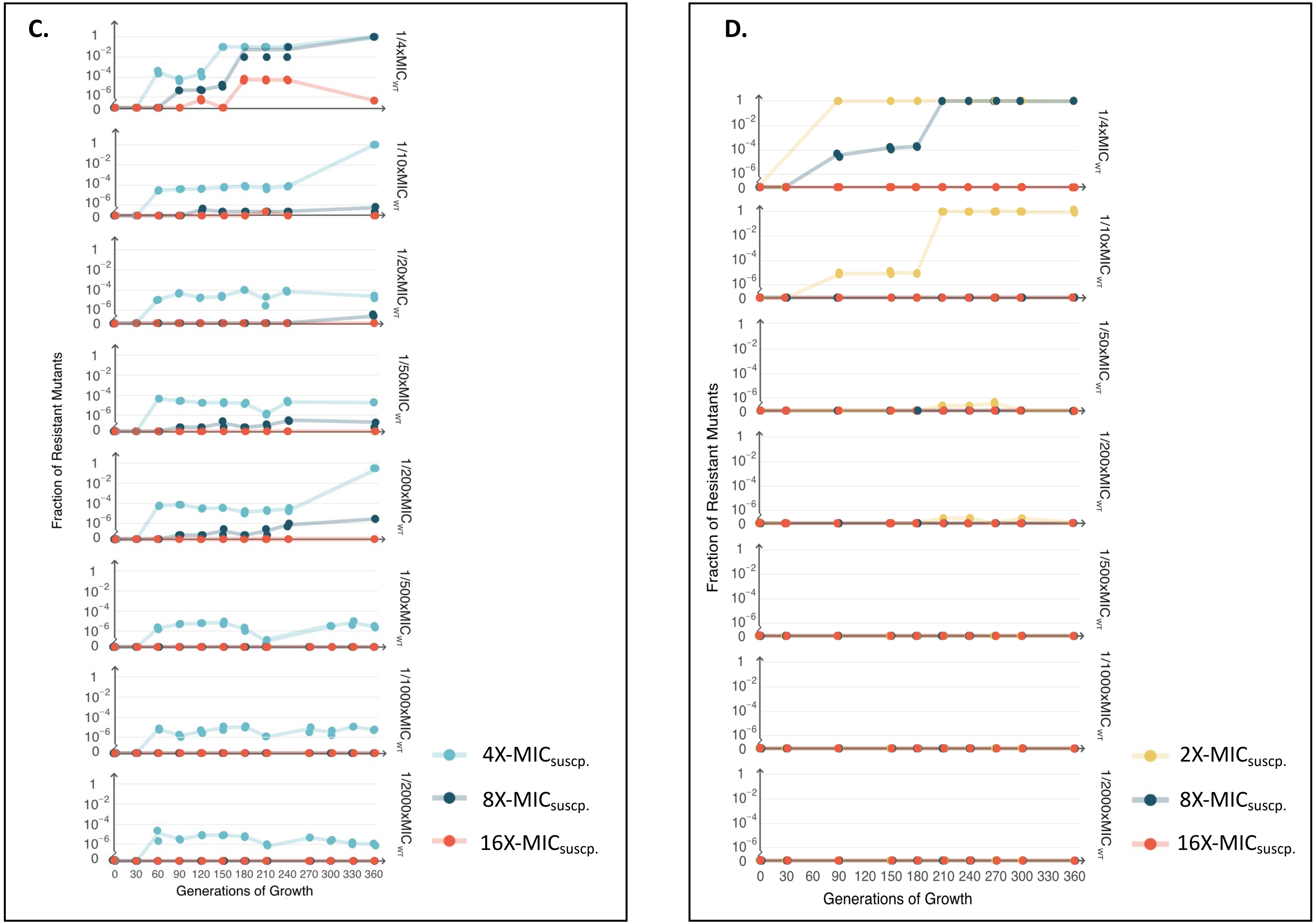

**Supplementary Fig. 2**. Exponential growth-rates at different antibiotic concentrations for resistant mutants isolated on A) Fosfomycin B) Tetracycline C) Nitrofurantoin and D) Ciprofloxacin

**A.**

Mutants isolated at 1/4$^{th}$ MIC$_{susceptible}$ of fosfomycin

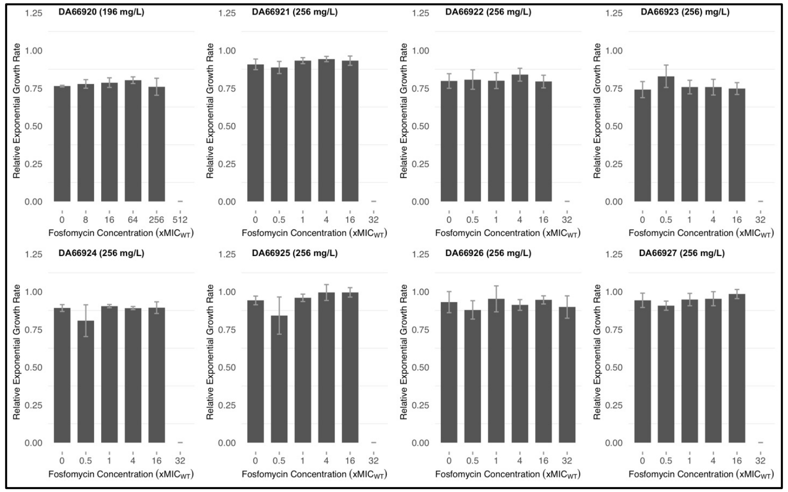


Mutants isolated at 1/200$^{th}$ MIC$_{susceptible}$ of fosfomycin

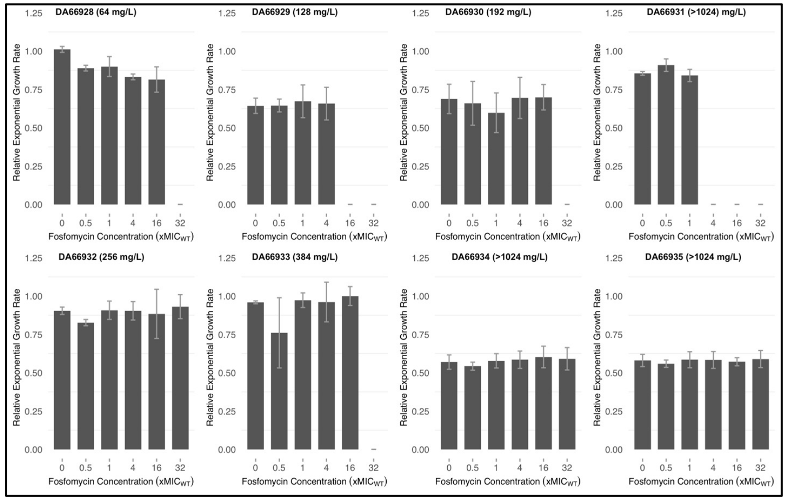


Mutants isolated at 1/2000$^{th}$ MIC$_{susceptible}$ of fosfomycin

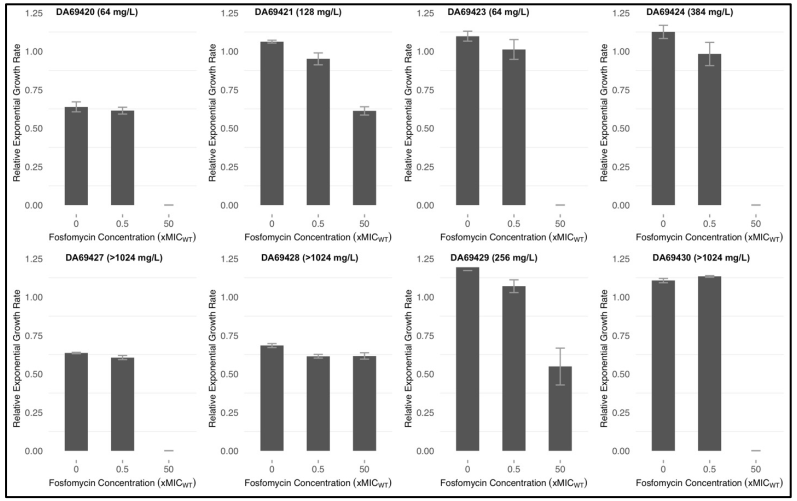


**B.**

Mutants isolated at 1/4$^{th}$ MIC$_{susceptible}$ of tetracycline

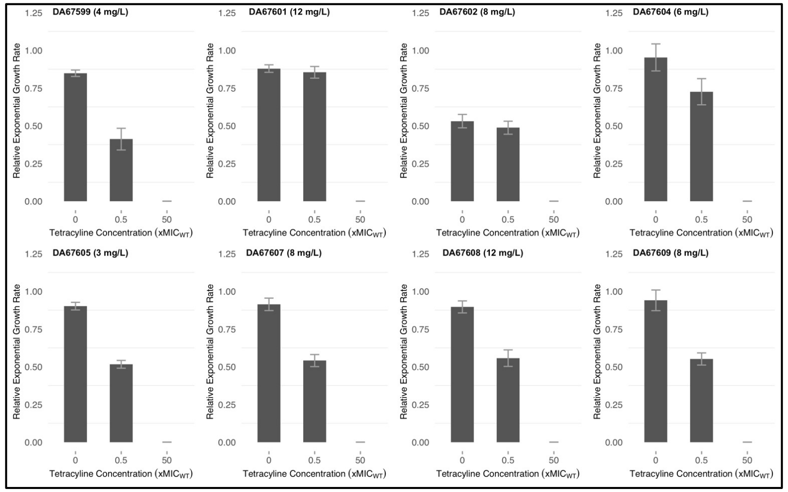


Mutants isolated at 1/200$^{th}$ MIC$_{susceptible}$ of tetracycline

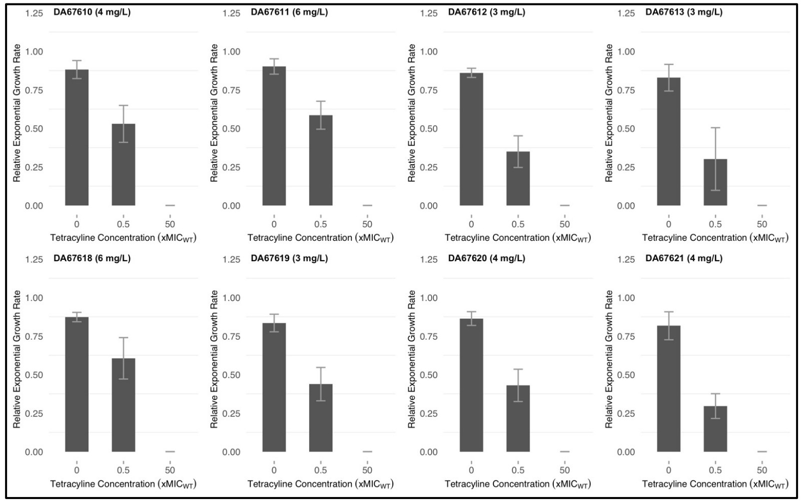

**Supplementary Fig. 2**. Exponential growth-rates at different antibiotic concentrations for resistant mutants isolated on A) Fosfomycin B) Tetracycline C) Nitrofurantoin and D) Ciprofloxacin

**C.**

Mutants isolated at 1/4th $MIC_{susceptible}$ of nitrofurantoin

Mutants isolated at 1/200th $MIC_{susceptible}$ of nitrofurantoin

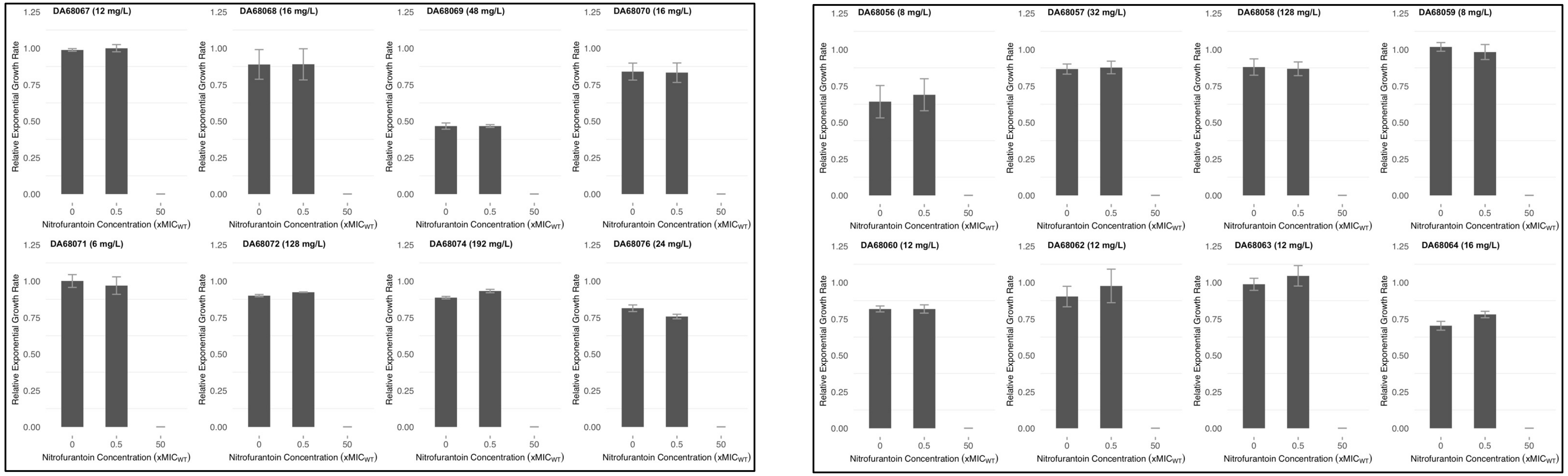


**D.**

Mutants isolated at 1/4th $MIC_{susceptible}$ of ciprofloxacin

Mutants isolated at 1/10th $MIC_{susceptible}$ of ciprofloxacin

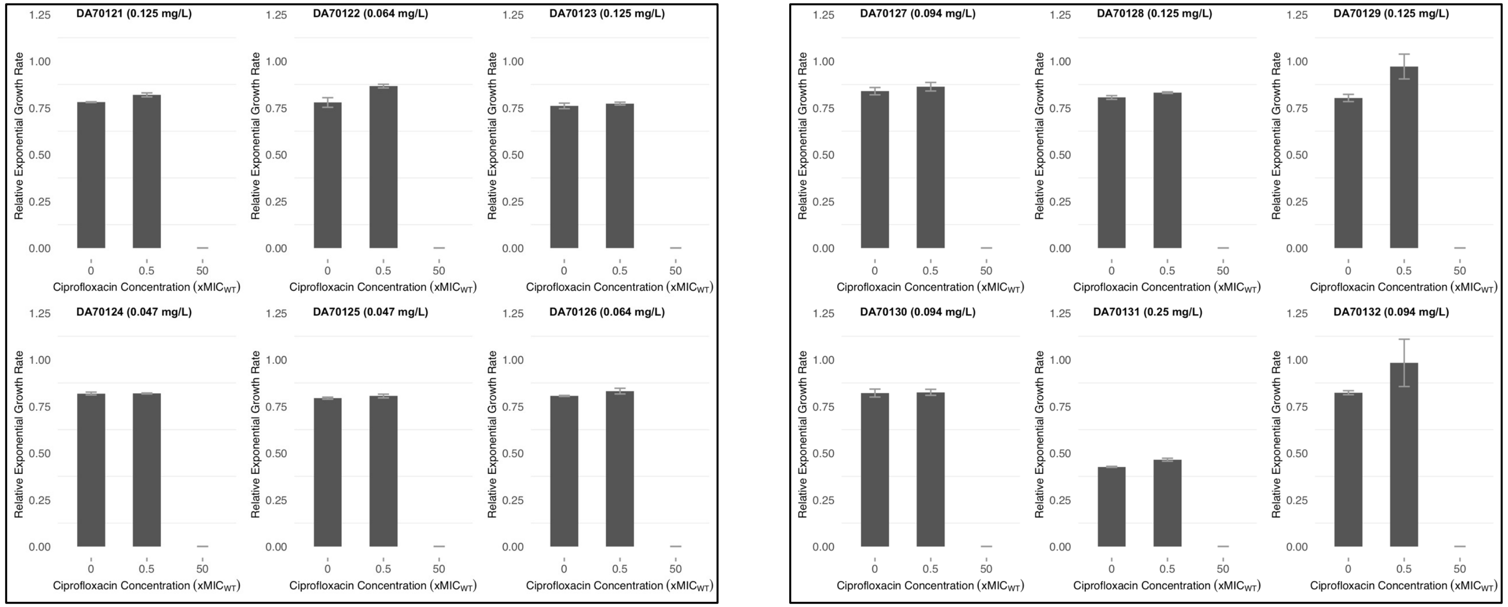

**Supplementary Fig. 3.** Mutations identified through whole-genome sequencing of resistant mutants isolated at the end-point of the experiment for populations evolving at sub-MICs of A) Fosfomycin B) Tetracycline C) Nitrofurantoin and D) Ciprofloxacin. Genes marked green are known resistance genes for the antibiotic on which selection was performed, those marked in yellow are known resistance genes but for other antibiotics and those marked red have been shown to confer media adaptation.

## A.

| Sel. | Strain | *glpT* | *ptsP* | *uhpT* | *fimE* | *kdpD* | *rph* | *ydcI* | *mdtF* | *ubiF* | *uhpC* | *yehH* | *yeaI* | *iscR* | *uhpA* | *trkH* | *nmpC* |
|---|---|---|---|---|---|---|---|---|---|---|---|---|---|---|---|---|---|
| Sel.: 1/4xMIC | DA66920 | c.264A>T D88E | c.1018G>A Q340* | c.214::IS1 | c214:: IS1 | | | | | | | | | | | | |
| | DA66921 | c.862:: IS5 | | c.1093A>G F365L | | c.1523C>T R508H | c.619C>A E507* | c.21:: IS1 | c.1402:: IS1 | | | | | | | | |
| | DA66922 | c.862:: IS5 | | | c.125:: IS5 | c.2321T>C D774G | | c.772T>A K258* | | c.705C>G R235= | c.214T>G T72P | | | | | | |
| | DA66923 | c.862:: IS5 | | | c.125:: IS5 | c.2321T>C D774G | | c.85:: IS2 | | c.705C>G R235= | c.214T>G T72P | c.760T>C V254A | | | | | |
| | DA66924 | c.275del V86 fs | | | | c.2507C>T G836E | | c.230T>C H77R | | | | | c.1029C>A F343L | c.182G>A P61L | c.130_139del Δ L43-G45 | | |
| | DA66925 | c.264A>T D88E | | c.948:: IS186 | c.272:: IS5 | | | | | | | | | | | c.239T>A L80K | |
| | DA66926 | c.274 L255R | | | | c.1859C>A R620L | | | | | | | | | | | c.1040_ 2031del Δ L1-A337 |
| | DA66927 | c.264A>T D88E | | c.948:: IS186 | c.272:: IS5 | | | c.698G>A T233M | | | | | | | | c.239T>A L80K | |
| Sel.: 1/200xMIC | DA66928 | | | c.1257_ 1262del P423 fs | | | c.609_691del Δ A203-R230 | | | | | | | | | | |
| | DA66929 | | | | c.125:: IS5 | c.1532C>A R511L | | c.171del Q58 fs | c.1402:: IS1 | | c.235:: IS5 | | | | | | |
| | DA66930 | | | | c.125:: IS5 | c.1532C>A R511L | | c.171del Q58 fs | c.1402:: IS1 | | c.385C>T A129T | | | | | | |
| | DA66931 | | | | | c.1952T>G E651A | | c.5Tins F2 fs | | | | | | | | | |
| | DA66932 | | c.714del A239 fs | | | | | | | | | | | | | | |
| | DA66933 | c.632del P212 fs | c.714del A239 fs | | | | | c.241:: IS2 | | | | | | | | | |
| | DA66934 | | c.714del A239 fs | | | | | | | | c.1039G>A Q347* | | | | | | |
| | DA66935 | | | | c.201:: IS1 | | | | c.1402:: IS1 | | | | | | | c.387T>C Y130H | |
| Sel.: 1/2000xMIC | DA69420 | | | c.382::IS2 | | | | | | | | | | | | | |
| | DA69421 | c.909::IS5 | | | | | c.609_691del Δ A203-R230 | c.41C>A R14L | | | | | | | c.125_134del Δ A41-L43 | | |
| | DA69423 | | | | c.125:: IS5 | | c.609_691del Δ A203-R230 | c.395A>G L123S | | | c.760:: IS5 | | | | | | |
| | DA69424 | | | c.118::IS1 | c.6::IS1 | | c.609_691del Δ A203-R230 | | | | | | | | | | |
| | DA69427 | | | | c.483del G163 fs | c.1523C>T R508H | | | | | c.492G>T N164K | | | | | | |
| | DA69428 | | | | c.483del G163 fs | c.1523C>T R508H | | c.275del S92 fs | | | c.360_401del Δ Q131-S143 | | | | | | |
| | DA69429 | c.1302_ 1347del Δ I443-R456 | | | c.519:: IS5 | | c.609_691del Δ A203-R230 | | | | | | | | c.125_134del Δ A41-L43 | | |
| | DA69430 | | | | c.519:: IS5 | | c.609_691del Δ A203-R230 | | | | | | | | c.125_134del Δ A41-L43 | | |

| Sel. | Strain | *aat* | *yffS* | *rbsR* | *hchA* | *uhpB* | *bglG* | *ymdA* | *ydeJ* | *ptsI* | *allB* | *putP* | *yegX* | *sapC* | *puuB* | *nhaR* | *yobF* | *gatA* | *yphG* | *gatB* | Intergenic insertions | Deletions |
|---|---|---|---|---|---|---|---|---|---|---|---|---|---|---|---|---|---|---|---|---|---|---|
| Sel.: 1/4xMIC | DA66920 | | | | | | | | | | | | | | | | | | | | | |
| | DA66921 | | | | | | | | | | | | | | | | | | | | | |
| | DA66922 | | | | | | | | | | | | | | | | | | | | | |
| | DA66923 | | | | | | | | | | | | | | | | | | | | | |
| | DA66924 | | | | | | | | | | | | | | | | | | | | | |
| | DA66925 | | | | | | | | | | | | | | | | | | | | | Δ [*trg*]-[*ydcI*] (1.163kbp) |
| | DA66926 | | | | | | | | | | | | | | | | | | | | *uhpT*(58)/*uhpC*(-72)::IS1<br>*nrfG*(-227)/*gltP*(115):A>T<br>*fimE*(-363)/*fimA*(119)insA<br>*flhD*(400)/*uspC*(373)::IS1 | Δ *trg-yncG* (33.089kbp) |
| | DA66927 | | | | | | | | | | | | | | | | | | | | | |
| Sel.: 1/200xMIC | DA66928 | c.437C>T G146D | c.442G>A A148T | c.526G>A A181T | | | | | | | | | | | | | | | | | *fimE*(-198)/*fimA*(281)::IS5<br>*lrhA*(60)/*yfbQ*(852)::IS1 | |
| | DA66929 | | | | c.635:: IS1 | | | | | | | | | | | | | | | | *nrfG*(-227)/*gltP*(115):A>T<br>*flhD*(282)/*uspC*(498)::IS2 | |
| | DA66930 | | | | c.635:: IS1 | | | c.176del G59 fs | | | | | | | | | | | | | *flhD*(282)/*uspC*(498)::IS2 | |
| | DA66931 | | | | | c.99_100del V33 fs | c.245A>C L82* | | | | | | | | | | | | | | | |
| | DA66932 | | | c.360:: IS2 | | c.395C>T W132* | | | | | | | | | | | | | | | *flhD*(591)/*uspC*(182)::IS1<br>*nrfG*(-227)/*gltP*(115):A>T | |
| | DA66933 | | | c.360:: IS2 | | c.1405C>T G469R | | c.289:: IS2 | c.29:: IS2 | | | | | | | | | | | | | *fimE*(-354)/*fimA*(128): Δ1bp |
| | DA66934 | | | c.360:: IS2 | | | | | | c.1476_ 1478del H493 fs | | | | | | | | | | | *ygjH*(39)/*ebgR*(180): C>A<br>*flhD*(591)/*uspC*(182)::IS1<br>*nrfG*(-227)/*gltP*(115):A>T | *fimE*(-354)/*fimA*(128): Δ1bp |
| | DA66935 | | | | | c.1012::IS1 | | | | | c.735C>A S245R | c.179T>A L60Q | | | | | | | | | *flhD*(315)/*uspC*(457)::IS1 | |
| Sel.: 1/2000xMIC | DA69420 | | | | | | | | | | | | c.574G>T P192Q | | | | | | | | *wza*(250)/*yegH*(409):G>A<br>*fimB*(-461)/*fimE*(14)::IS5<br>*flhD*(605)/*uspC*(171)::IS2 | Δ *ykfJ-ykfC* (19.892kbp) IS5-mediated<br>*pyrE*(39)/*rph*(-55): Δ2bp |
| | DA69421 | | | | | | | | | | | | | | | | | | | | *fimE*(-198)/*fimA*(281)::IS5<br>*flhD*(605)/*uspC*(171)::IS2 | |
| | DA69423 | | | | | | | | | | | | | | | | | | | | *hns*(274)/*tdk* (328)::IS5<br>*ycaC*(168)/*ycaD* (138)::IS1<br>*flhD*(513)/*uspC*(264)::IS5 | |
| | DA69424 | | | | | | | | | | | | | c.193G>T P65T | c.762C>A G254= | c.370_ 1082del ΔI124-C360 | | | | | | |
| | DA69427 | | | | | | | | | | | | | | | | c.56C>G R19P | c.203: :IS5 | c.2550T>C L750= | | | Δ *gpt-ykfC* (17.278kbp) IS5-mediated |
| | DA69428 | | | | | | | | | | | | | | | | | | c.2550T>C L750= | c.68:: IS5 | | |
| | DA69429 | | | c.506C>T T169M | | | | | | | | | | | | | | | | | *flhD*(657)/*uspC*(120)::IS5 | |
| | DA69430 | | | c.506C>T T169M | | | | | | | | | | | | | | | | | *flhD*(657)/*uspC*(120)::IS5 | |

* stop codon; Δ deletion; fs frame shift; del deletion; ins insertion; dup duplication; c. coding region

- Green: Genes mutated in our study that earlier have been described to confer resistance to fosfomycin
- Red: Genes mutated in our study that earlier have been described as genetic adaptations to growth in laboratory conditions (Knöppel et al., 2018)
- Yellow: Genes mutated in our study that earlier have been described to confer antimicrobial resistance

**Supplementary Fig. 3.** Mutations identified through whole-genome sequencing of resistant mutants isolated at the end-point of the experiment for populations evolving at sub-MICs of A) Fosfomycin B) Tetracycline C) Nitrofurantoin and D) Ciprofloxacin. Genes marked green are known resistance genes for the antibiotic on which selection was performed, those marked in yellow are known resistance genes but for other antibiotics and those marked red have been shown to confer media adaptation.

**B.**

| | | *envZ* | *fbaA* | *malT* | *yahL* | *gatY* | *yeaR* | *mdtF* | *rho* | *rpoC* | *fimE* | *gatC* | *elaD* | *dhaK* | *plsB* | *trkA* | *ptsP* | *aat* | *ymdA* | *kdpD* | *suhB* | *mutL* | *mutS* | Intergenic insertions |
|---|---|---|---|---|---|---|---|---|---|---|---|---|---|---|---|---|---|---|---|---|---|---|---|---|
| Sel.: 1/4xMIC | DA67599 | c.66C>A<br>L22F | c.461T>C<br>N154S | c.658A>C<br>T220P | c.527::<br>IS5 | c.644::<br>IS5 | c.115::<br>IS186 | c.1402::<br>IS1 | c.259C>T<br>R87C | | | | | | | | | | | | | | | *malP*(460)/*malT*(148)::IS5 |
| | DA67601 | c.40G>A<br>R14C | | | | | | | | c.2176G>A<br>A726T<br>c.3149A>G<br>T1050A | | | | | | | | | | | | | c.653G>A<br>G218E<br>c.2444::IS1 | |
| | DA67602 | c.40G>A<br>R14C | c.704A>G<br>V235A | | | | | | | | c.501::<br>IS1 | | | | | | | | | | | | c.2444::IS1 | |
| | DA67604 | c.442G>A<br>P148S | | | | | | | c.250A>T<br>S84C | | c.168::<br>IS1 | | | | | | | | | | | | | |
| | DA67605 | | | | | | | | | | c.272::<br>iS5 | c.85::<br>IS5 | | | | | | | | | | | | |
| | DA67607 | | | | | | | | | | | | c.963C>A<br>A321= | | | | | | | | | | | |
| | DA67608 | c.1189G>A<br>R397C | | | | | | | | | c.272::<br>iS5 | c.85::<br>IS5 | | c.858G>C<br>G268= | c.794C>A<br>R265L | c.1078A>C<br>S360R | | | | | | | | *ompF*(220)/*ansS*(-383):A>G |
| | DA67609 | c.1189G>A<br>R397C | | | | | | | | | c.272::<br>iS5 | c.85::<br>IS5 | | | c.794C>A<br>R265L | | | | | | | | | |
| Sel.: 1/200xMIC | DA67610 | c.146A>G<br>V49A | | | | | | c.2297T>C<br>V766A | | | c.125::<br>IS5 | c.626::<br>IS5 | | | | | c.2002A>G<br>C668R | c.571T>C<br>N191D | c.303::IS5 | c.823A>G<br>S275P | | c.275T>C<br>L92P | | |
| | DA67611 | | | | | | | c.1402::<br>IS1 | | | | | | | | | | | c.48delT | | | | | |
| | DA67612 | | | | | | | | | | c.125::<br>IS5 | c.626::<br>IS5 | | | | | c.2002A>G<br>C668R | | c.309::IS5 | | | c.275T>C<br>L92P | | |
| | DA67613 | | | | | | | c.1402::<br>IS1 | | | | | | | | | c.1063A>G<br>S355P | | c.309::IS5 | c.986A>G<br>V329A | | | c.1966C>A<br>R656S | |
| | DA67618 | | | | | | | | | | c.125::<br>IS5 | | | | | | | | c.309::IS5 | | c.699<br>G233= | | | |
| | DA67619 | c.146A>G<br>V49A | c.551T>C<br>D184G | | | | | | | | c.125::<br>IS5 | c.626::<br>IS5 | | | | | c.2002A>G<br>C668R | c.571T>C<br>N191D | c.309::IS5 | | | c.275T>C<br>L92P | | |
| | DA67620 | c.146A>G<br>V49A | c.551T>C<br>D184G | | | | | | | | c.125::<br>IS5 | c.626::<br>IS5 | | | | | c.2002A>G<br>C668R | c.571T>C<br>N191D | c.309::IS5 | c.823A>G<br>S275P | | c.275T>C<br>L92P | | |
| | DA67621 | | | | | | | | | | c.125::<br>IS5 | c.626::<br>IS5 | | | | | | | c.309::IS5 | | | c.275T>C<br>L92P | | |

Positions for mutations outside of coding sequences are given relative to the first nucleotide in the start codon of the downstream gene (negative numbers).

* stop codon; Δ deletion; fs frame shift; del deletion; ins insertion; dup duplication; c. coding region

Genes mutated in our study that earlier have been described to confer resistance to tetracycline

Genes mutated in our study that earlier have been described as genetic adaptations to growth in laboratory conditions (Knöppel et al., 2018)

Genes mutated in our study that earlier have been described to confer antimicrobial resistance

Strains in our study that were found to be hypermutators

**Supplementary Fig. 3.** Mutations identified through whole-genome sequencing of resistant mutants isolated at the end-point of the experiment for populations evolving at sub-MICs of A) Fosfomycin B) Tetracycline C) Nitrofurantoin and D) Ciprofloxacin. Genes marked green are known resistance genes for the antibiotic on which selection was performed, those marked in yellow are known resistance genes but for other antibiotics and those marked red have been shown to confer media adaptation.

**C.**

| | | *nfsA* | *yeaR* | *uhpT* | *relA* | *yfaS* | *nfsB* | *ptwF* | *ycgB* | *menC* | *leuV* | *rpsK* | *fimE* | *kdpD* | *rph* | *mpaA* | *ydcI* | *yhaC* | *mutT* | *yfdE* | *cheA* | *ptsP* | *yobF* | *putP* | *aat* | *mdtH* | *degQ* | *rho* | Intergenic insertions | Deletions |
|---|---|---|---|---|---|---|---|---|---|---|---|---|---|---|---|---|---|---|---|---|---|---|---|---|---|---|---|---|---|---|
| Sel.: 1/4xMIC | DA68067 | c.674:: IS30 | c.115:: IS186 | c.1003_1281del Δ A335-G427 | c.2172_2184del Δ A274-G727 | | | | | | | | | | | | | | | | | | | | | | | | *dut*(-84)/*slmA* (23):T>C | |
| | DA68068 | c.674:: IS30 | c.115:: IS186 | c.1003_1281del Δ A335-G427 | c.2172_2184del Δ A274-G727 | c.902G>A T30I | | | | | | | | | | | | | | | | | | | | | | | *dut*(-84)/*slmA* (23):T>C | |
| | DA68069 | c.380:: IS186 | | | | | c.38:: IS5 | c.1C>A R1= | c.192:: IS2 | c.132:: IS196 | c.65:: IS186 | c.116C>T G39D | | | | | | | | | | | | | | | | | *lhrA*(137)/*yfbQ* (780)::IS5 | |
| | DA68070 | c.674:: IS30 | c.115:: IS186 | c.1003_1281del Δ A335-G427 | c.2172_2184del Δ A274-G727 | | | | | | | | | | | | | | | | | | | | | | | | *dut*(-84)/*slmA* (23):T>C | |
| | DA68071 | c.674:: IS30 | | c.1003_1281del Δ A335-G427 | c.2172_2184del Δ A274-G727 | | | | | | | | | | | | | | | | | | | | | | | | *dut*(-84)/*slmA* (23):T>C | |
| | DA68072 | c.380:: IS186 | | | | | c.38:: IS5 | | | | | | | | | | | | | | | | | | | | | | | |
| | DA68074 | c.380:: IS186 | c.115:: IS186 | | | | c.38:: IS5 | | | | | | c.125:: IS5 | | | | | | | | | | | | | | | | | |
| | DA68076 | c.674:: IS30 | c.115:: IS186 | c.1003_1281del Δ A335-G427 | c.2172_2184del Δ A274-G727 | | | | | | | | | | | | | | | | | | | | | | | | *dut*(-84)/*slmA* (23):T>C | |
| Sel.: 1/200xMIC | DA68056 | c.289del L98 fs | | | | | | | | | | | | c.2564A>C V845G | c.609_691del Δ A203-R230 | c.555A>C D185E | c.5del N4 fs | c.1101:: IS5 | | | | | | | | | | | *csgC*(-54)/*ymdA* (63)::IS2 | |
| | DA68057 | c.434T>G L145 * | | | | | | | | | | | c.272:: IS5 | | | | | | c.68G>T R23L | | | c.1274A>C L425* | | | | | | | | |
| | DA68058 | c.635G>A W212* | | | | | c.38:: IS5 | | | | | | c.259A>C T87P | | | | | | | c.457:: IS2 | c.125G>A A42V | c.66del N23 fs | | | | | | | | Δ *ymdE-[ymdC]* (12.112kbp) IS3-mediated *pyrE*(42)/*rph*(-53):: Δ1bp |
| | DA68059 | | | | | | | | | | | | | c.1621A>G S541P | c.609_691del Δ A203-R230 | | | | | | | | c.106:: IS1 | | | | | | *fimB*(-461)/*fimE*(14)::IS5 | |
| | DA68060 | | | | | | | c.1C>A R1= | | | | | c.218A>C H73P | C.1277G>A A426V | c.609_691del Δ A203-R230 | | | | | | | | | c.1012G>T V338L | c.125:: IS1 | | | | *ompC*(128)/*rcsD* (608)::IS5 | Δ *yrdA-yrdD* (562bp) IS1-mediated |
| | DA68062 | c.380:: IS186 | | | | | | | | | | | c.259A>C T87P | | | | | | | | c.125G>A A42V | c.66del N23 fs | | | | c.296G>T P99Q | c.356T>C I119T | | yjhY(51)/*yjhP* (-76):T>A | Δ *ymdE-[ymdC]* (12.112kbp) IS3-mediated *pyrE*(42)/*rph*(-53):: Δ1bp |
| | DA68063 | c.380:: IS186 | | | | | | c.1C>A R1= | | | | | c.217A>C H73P | c.1277G>A A426V | c.609_691del Δ A203-R230 | | | | | | | | | c.1012G>T V338L | c.125:: IS1 | | | | | |
| | DA68064 | c.380:: IS186 | | | | | | | | | | | | c.1898G>A T633M | | | c.278:: IS2 | | | | | | | | | | | c.304C>A R102S | *fimE*(-198)/*fimA*(281)::IS5 | Δ *marA-marB* (94bp) |

* stop codon; Δ deletion; fs frame shift; del deletion; ins insertion; dup duplication; c. coding region

Genes mutated in our study that earlier have been described to confer resistance to nitrofurantoin

Genes mutated in our study that earlier have been described as genetic adaptations to growth in laboratory conditions (Knöppel et al., 2018)

Genes mutated in our study that earlier have been described to confer antimicrobial resistance

Strains in our study that were found to be hypermutators

**Supplementary Fig. 3.** Mutations identified through whole-genome sequencing of resistant mutants isolated at the end-point of the experiment for populations evolving at sub-MICs of A) Fosfomycin B) Tetracycline C) Nitrofurantoin and D) Ciprofloxacin. Genes marked green are known resistance genes for the antibiotic on which selection was performed, those marked in yellow are known resistance genes but for other antibiotics and those marked red have been shown to confer media adaptation.

**D.**

| | | *ydcI* | *gyrB* | *ptsP* | *icd* | *emrA* | *yphG* | *ssuD* | *rph* | *yhaI* | *trkH* |
|---|---|---|---|---|---|---|---|---|---|---|---|
| Sel.: 1/4xMIC | DA70121 | c.859_894del<br>Δ D286-I298 | c.1389_1394del<br>A65 fs | c.1545_1555del<br>R119 fs | c.1098C>T H366=<br>c.1123T>C L375= | c.591A>T<br>P197= | c.2250T>C<br>L750= | | | | |
| | DA70122 | | c.1389_1394del<br>A65 fs | | c.1098C>T H366=<br>c.1100C>T T370=<br>c.1123T>C L375=<br>c.1155C>T N385=<br>c.1158G>C A386=<br>c.1161A>G K387= | | c.2250T>C<br>L750= | c.703T>C<br>E243= | c.559_560del<br>S186 fs | c.68::<br>IS1 | c.1040C>A<br>A347D |
| | DA70123 | | | c.1545_1555del<br>R119 fs | | | | | | | |
| | DA70124 | c.735_744del<br>Δ E79-Y248 | | c.1545_1555del<br>R119 fs | | | | | | | |
| | DA70129 | | | c.328_330dup<br>L111 fs | | | | | | | |
| | DA70130 | | | c.328_330dup<br>L111 fs | | | | | | | |
| | DA70132 | | | c.328_330dup<br>L111 fs | | | | | | | |
| Sel.: 1/10xMIC | DA70133 | | c.1389_1394del<br>A65 fs | | c.1098C>T H366=<br>c.1100C>T T370=<br>c.1123T>C L375= | | c.2250T>C<br>L750= | | | | |
| | DA70134 | | c.1389_1394del<br>A65 fs | | c.1098C>T H366=<br>c.1100C>T T370=<br>c.1123T>C L375= | | c.2250T>C<br>L750= | | | | |
| | DA70135 | | | c.1678_1681del<br>T560 fs | | | | | c.609_691del<br>Δ A203-R230 | | |
| | DA70137 | | | | | | | | | | |

| | | *gyrA* | *ymdA* | *caiD* | *fimE* | *brnQ* | *ydhL* | *tap* | *proB* | *kdpD* | *ydcT* | *cho* | *ydeO* | *oxyR* | *ssuA* | *rpsF* | Intergenic insertions | Deletions |
|---|---|---|---|---|---|---|---|---|---|---|---|---|---|---|---|---|---|---|
| Sel.: 1/4xMIC | DA70121 | | | | | | | | | | | | | | | | | |
| | DA70122 | | | | | | | | | | | | | | | | *yaiS*(547)/*tauA* (66)::IS5<br>*rrlE*(-13)/*rrfE*(81):G>C | *rrlE*(-27)/*rrfE*(67): Tdel |
| | DA70123 | c.242C>T<br>G81D | c.309::<br>IS5 | c.500T>G<br>E167A | c.125::<br>IS5 | | | | | | | | | | | | *pyrE*(46)/*rph*(-49):C>T<br>*gltT*(-171)/*rrlB*(23):A>T | |
| | DA70124 | c.247_249del<br>S96 fs | | | | c.1256_1266del<br>Δ M419-L422 | c.188C>T<br>R63H | | | | | | | | | | *mipA*(358)/*yeaG* (74)::IS2<br>*ykgD*(-427)/*ykgE*(97)::IS5<br>*cirA*(230)/*lysP*(-64):G>T | Δ (*uspC otsA [otsB]*)<br>(2.153bp) |
| | DA70129 | c.242C>T<br>G81D | c.84del<br>F18 fs | | c.125::<br>IS5 | | | c.822_832dup | | | | | | | | | *pyrE*(46)/*rph*(-49):C>T<br>*gltT*(-171)/*rrlB*(23):A>T | |
| | DA70130 | c.242C>T<br>G81D | c.309::<br>IS5 | | c.125::<br>IS5 | | | | c.352insCC<br>R118 fs | | | | | | | | *pyrE*(46)/*rph*(-49):C>T<br>*gltT*(-171)/*rrlB*(23):A>T<br>yfjL(310)/yfjM(-<br>50):insTARGGCAC | |
| | DA70132 | c.242C>T<br>G81D | c.309::<br>IS5 | | c.125::<br>IS5 | | | | c.352insCC<br>R118 fs | | | | | | | | *pyrE*(46)/*rph*(-49):C>T<br>*gltT*(-171)/*rrlB*(23):A>T<br>yfjL(310)/yfjM(-<br>50):insTARGGCAC | |
| Sel.: 1/10xMIC | DA70133 | | | | | | | | | c.1523C>T<br>R508H | c.33G>T<br>S11= | | | | | | *pyrE*(40)/*rph*(-55):delA<br>*pflA*(188)/*pflB*(-4):C>T | |
| | DA70134 | | | | | | | | | c.1523C>T<br>R508H | | | | | | | *fimE*(-363)/*fimA*(119):insA | |
| | DA70135 | c.247_249del<br>D40 fs | | | | | | | | | | c.391G>A<br>A131T | c.7C>A<br>S2= | c.572_585del<br>L191 fs | | | *pspF*(96)/*pspA* (71): A>G | Δ *intS-pawZ* (10.2015kbp)<br>Δ ([ydcI] [ydcJ]) (1566bp) |
| | DA70137 | c.260T>C<br>D87G | | | | | | | | | | | | | c.710T>C<br>D337G | c.349_350del<br>A116 fs | *fimB*(-461)/*fimE*(14)::IS5 | |

* stop codon; Δ deletion; fs frame shift; del deletion; ins insertion; dup duplication; c. coding region

(green) Genes mutated in our study that earlier have been described to confer resistance to ciprofloxacin

(red) Genes mutated in our study that earlier have been described as genetic adaptations to growth in laboratory conditions (Knöppel et al., 2018)

(yellow) Genes mutated in our study that earlier have been described to confer antimicrobial resistance

## Supplementary Table 1. List of strains used in this study.

| Strain | Reference/source | Comment |
|---|---|---|
| DA4201 | Strain collection | *E. coli* MG1655 Wild type (parental) |
| DA66920 | This study | *E. coli* mutant isolated from evolution in presence of fosfomycin at concentration of 1/4 the MIC of the WT strain. |
| DA66921 | This study | *E. coli* mutant isolated from evolution in presence of fosfomycin at concentration of 1/4 the MIC of the WT strain. |
| DA66922 | This study | *E. coli* mutant isolated from evolution in presence of fosfomycin at concentration of 1/4 the MIC of the WT strain. |
| DA66923 | This study | *E. coli* mutant isolated from evolution in presence of fosfomycin at concentration of 1/4 the MIC of the WT strain. |
| DA66924 | This study | *E. coli* mutant isolated from evolution in presence of fosfomycin at concentration of 1/4 the MIC of the WT strain. |
| DA66925 | This study | *E. coli* mutant isolated from evolution in presence of fosfomycin at concentration of 1/4 the MIC of the WT strain. |
| DA71962 | This study | *E. coli* mutant isolated from evolution in presence of fosfomycin at concentration of 1/4 the MIC of the WT strain. |
| DA71963 | This study | *E. coli* mutant isolated from evolution in presence of fosfomycin at concentration of 1/4 the MIC of the WT strain. |
| DA66926 | This study | *E. coli* mutant isolated from evolution in presence of fosfomycin at concentration of 1/4 the MIC of the WT strain. |
| DA71964 | This study | *E. coli* mutant isolated from evolution in presence of fosfomycin at concentration of 1/4 the MIC of the WT strain. |
| DA66927 | This study | *E. coli* mutant isolated from evolution in presence of fosfomycin at concentration of 1/4 the MIC of the WT strain. |
| DA71965 | This study | *E. coli* mutant isolated from evolution in presence of fosfomycin at concentration of 1/4 the MIC of the WT strain. |
| DA66928 | This study | *E. coli* mutant isolated from evolution in presence of fosfomycin at concentration of 1/200 the MIC of the WT strain. |
| DA71966 | This study | *E. coli* mutant isolated from evolution in presence of fosfomycin at concentration of 1/200 the MIC of the WT strain. |
| DA66929 | This study | *E. coli* mutant isolated from evolution in presence of fosfomycin at concentration of 1/200 the MIC of the WT strain. |
| DA66930 | This study | *E. coli* mutant isolated from evolution in presence of fosfomycin at concentration of 1/200 the MIC of the WT strain. |
| DA66931 | This study | *E. coli* mutant isolated from evolution in presence of fosfomycin at concentration of 1/200 the MIC of the WT strain. |
| DA71967 | This study | *E. coli* mutant isolated from evolution in presence of fosfomycin at concentration of 1/200 the MIC of the WT strain. |
| DA66932 | This study | *E. coli* mutant isolated from evolution in presence of fosfomycin at concentration of 1/200 the MIC of the WT strain. |
| DA71968 | This study | *E. coli* mutant isolated from evolution in presence of fosfomycin at concentration of 1/200 the MIC of the WT strain. |
| DA66933 | This study | *E. coli* mutant isolated from evolution in presence of fosfomycin at concentration of 1/200 the MIC of the WT strain. |
| DA66934 | This study | *E. coli* mutant isolated from evolution in presence of fosfomycin at concentration of 1/200 the MIC of the WT strain. |
| DA66935 | This study | *E. coli* mutant isolated from evolution in presence of fosfomycin at concentration of 1/200 the MIC of the WT strain. |
| DA71969 | This study | *E. coli* mutant isolated from evolution in presence of fosfomycin at concentration of 1/200 the MIC of the WT strain. |
| DA69420 | This study | *E. coli* mutant isolated from evolution in presence of fosfomycin at concentration of 1/2000 the MIC of the WT strain. |
| DA69421 | This study | *E. coli* mutant isolated from evolution in presence of fosfomycin at concentration of 1/2000 the MIC of the WT strain. |
| DA69422 | This study | *E. coli* mutant isolated from evolution in presence of fosfomycin at concentration of 1/2000 the MIC of the WT strain. |
| DA69423 | This study | *E. coli* mutant isolated from evolution in presence of fosfomycin at concentration of 1/2000 the MIC of the WT strain. |
| DA69424 | This study | *E. coli* mutant isolated from evolution in presence of fosfomycin at concentration of 1/2000 the MIC of the WT strain. |
| DA69425 | This study | *E. coli* mutant isolated from evolution in presence of fosfomycin at concentration of 1/2000 the MIC of the WT strain. |
| DA69426 | This study | *E. coli* mutant isolated from evolution in presence of fosfomycin at concentration of 1/2000 the MIC of the WT strain. |
| DA69427 | This study | *E. coli* mutant isolated from evolution in presence of fosfomycin at concentration of 1/2000 the MIC of the WT strain. |
| DA69428 | This study | *E. coli* mutant isolated from evolution in presence of fosfomycin at concentration of 1/2000 the MIC of the WT strain. |
| DA69429 | This study | *E. coli* mutant isolated from evolution in presence of fosfomycin at concentration of 1/2000 the MIC of the WT strain. |
| DA69430 | This study | *E. coli* mutant isolated from evolution in presence of fosfomycin at concentration of 1/2000 the MIC of the WT strain. |
| DA69431 | This study | *E. coli* mutant isolated from evolution in presence of fosfomycin at concentration of 1/2000 the MIC of the WT strain. |
| DA67598 | This study | *E. coli* mutant isolated from evolution in presence of tetracycline at concentration of 1/4 the MIC of the WT strain. |
| DA67599 | This study | *E. coli* mutant isolated from evolution in presence of tetracycline at concentration of 1/4 the MIC of the WT strain. |
| DA67600 | This study | *E. coli* mutant isolated from evolution in presence of tetracycline at concentration of 1/4 the MIC of the WT strain. |
| DA67601 | This study | *E. coli* mutant isolated from evolution in presence of tetracycline at concentration of 1/4 the MIC of the WT strain. |
| DA67602 | This study | *E. coli* mutant isolated from evolution in presence of tetracycline at concentration of 1/4 the MIC of the WT strain. |

| | | |
|---|---|---|
| DA67603 | This study | *E. coli* mutant isolated from evolution in presence of tetracycline at concentration of 1/4 the MIC of the WT strain. |
| DA67604 | This study | *E. coli* mutant isolated from evolution in presence of tetracycline at concentration of 1/4 the MIC of the WT strain. |
| DA67605 | This study | *E. coli* mutant isolated from evolution in presence of tetracycline at concentration of 1/4 the MIC of the WT strain. |
| DA67606 | This study | *E. coli* mutant isolated from evolution in presence of tetracycline at concentration of 1/4 the MIC of the WT strain. |
| DA67607 | This study | *E. coli* mutant isolated from evolution in presence of tetracycline at concentration of 1/4 the MIC of the WT strain. |
| DA67608 | This study | *E. coli* mutant isolated from evolution in presence of tetracycline at concentration of 1/4 the MIC of the WT strain. |
| DA67609 | This study | *E. coli* mutant isolated from evolution in presence of tetracycline at concentration of 1/4 the MIC of the WT strain. |
| DA67610 | This study | *E. coli* mutant isolated from evolution in presence of tetracycline at concentration of 1/200 the MIC of the WT strain. |
| DA67611 | This study | *E. coli* mutant isolated from evolution in presence of tetracycline at concentration of 1/200 the MIC of the WT strain. |
| DA67612 | This study | *E. coli* mutant isolated from evolution in presence of tetracycline at concentration of 1/200 the MIC of the WT strain. |
| DA67613 | This study | *E. coli* mutant isolated from evolution in presence of tetracycline at concentration of 1/200 the MIC of the WT strain. |
| DA67614 | This study | *E. coli* mutant isolated from evolution in presence of tetracycline at concentration of 1/200 the MIC of the WT strain. |
| DA67615 | This study | *E. coli* mutant isolated from evolution in presence of tetracycline at concentration of 1/200 the MIC of the WT strain. |
| DA67616 | This study | *E. coli* mutant isolated from evolution in presence of tetracycline at concentration of 1/200 the MIC of the WT strain. |
| DA67617 | This study | *E. coli* mutant isolated from evolution in presence of tetracycline at concentration of 1/200 the MIC of the WT strain. |
| DA67618 | This study | *E. coli* mutant isolated from evolution in presence of tetracycline at concentration of 1/200 the MIC of the WT strain. |
| DA67619 | This study | *E. coli* mutant isolated from evolution in presence of tetracycline at concentration of 1/200 the MIC of the WT strain. |
| DA67620 | This study | *E. coli* mutant isolated from evolution in presence of tetracycline at concentration of 1/200 the MIC of the WT strain. |
| DA67621 | This study | *E. coli* mutant isolated from evolution in presence of tetracycline at concentration of 1/200 the MIC of the WT strain. |
| DA68067 | This study | *E. coli* mutant isolated from evolution in presence of nitrofurantoin at concentration of 1/4 the MIC of the WT strain. |
| DA68068 | This study | *E. coli* mutant isolated from evolution in presence of nitrofurantoin at concentration of 1/4 the MIC of the WT strain. |
| DA68069 | This study | *E. coli* mutant isolated from evolution in presence of nitrofurantoin at concentration of 1/4 the MIC of the WT strain. |
| DA68070 | This study | *E. coli* mutant isolated from evolution in presence of nitrofurantoin at concentration of 1/4 the MIC of the WT strain. |
| DA68071 | This study | *E. coli* mutant isolated from evolution in presence of nitrofurantoin at concentration of 1/4 the MIC of the WT strain. |
| DA68072 | This study | *E. coli* mutant isolated from evolution in presence of nitrofurantoin at concentration of 1/4 the MIC of the WT strain. |
| DA68074 | This study | *E. coli* mutant isolated from evolution in presence of nitrofurantoin at concentration of 1/4 the MIC of the WT strain. |
| DA68075 | This study | *E. coli* mutant isolated from evolution in presence of nitrofurantoin at concentration of 1/4 the MIC of the WT strain. |
| DA68076 | This study | *E. coli* mutant isolated from evolution in presence of nitrofurantoin at concentration of 1/4 the MIC of the WT strain. |
| DA68077 | This study | *E. coli* mutant isolated from evolution in presence of nitrofurantoin at concentration of 1/4 the MIC of the WT strain. |
| DA68078 | This study | *E. coli* mutant isolated from evolution in presence of nitrofurantoin at concentration of 1/4 the MIC of the WT strain. |
| DA68056 | This study | *E. coli* mutant isolated from evolution in presence of nitrofurantoin at concentration of 1/200 the MIC of the WT strain. |
| DA68057 | This study | *E. coli* mutant isolated from evolution in presence of nitrofurantoin at concentration of 1/200 the MIC of the WT strain. |
| DA68058 | This study | *E. coli* mutant isolated from evolution in presence of nitrofurantoin at concentration of 1/200 the MIC of the WT strain. |
| DA68059 | This study | *E. coli* mutant isolated from evolution in presence of nitrofurantoin at concentration of 1/200 the MIC of the WT strain. |
| DA68060 | This study | *E. coli* mutant isolated from evolution in presence of nitrofurantoin at concentration of 1/200 the MIC of the WT strain. |
| DA68061 | This study | *E. coli* mutant isolated from evolution in presence of nitrofurantoin at concentration of 1/200 the MIC of the WT strain. |
| DA68062 | This study | *E. coli* mutant isolated from evolution in presence of nitrofurantoin at concentration of 1/200 the MIC of the WT strain. |
| DA68063 | This study | *E. coli* mutant isolated from evolution in presence of nitrofurantoin at concentration of 1/200 the MIC of the WT strain. |
| DA68064 | This study | *E. coli* mutant isolated from evolution in presence of nitrofurantoin at concentration of 1/200 the MIC of the WT strain. |
| DA68065 | This study | *E. coli* mutant isolated from evolution in presence of nitrofurantoin at concentration of 1/200 the MIC of the WT strain. |
| DA68066 | This study | *E. coli* mutant isolated from evolution in presence of nitrofurantoin at concentration of 1/200 the MIC of the WT strain. |
| DA70121 | This study | *E. coli* mutant isolated from evolution in presence of ciprofloxacin at concentration of 1/4 the MIC of the WT strain. |
| DA70122 | This study | *E. coli* mutant isolated from evolution in presence of ciprofloxacin at concentration of 1/4 the MIC of the WT strain. |
| DA70123 | This study | *E. coli* mutant isolated from evolution in presence of ciprofloxacin at concentration of 1/4 the MIC of the WT strain. |
| DA70124 | This study | *E. coli* mutant isolated from evolution in presence of ciprofloxacin at concentration of 1/4 the MIC of the WT strain. |

| | | |
|---|---|---|
| DA70125 | This study | *E. coli* mutant isolated from evolution in presence of ciprofloxacin at concentration of 1/4 the MIC of the WT strain. |
| DA70126 | This study | *E. coli* mutant isolated from evolution in presence of ciprofloxacin at concentration of 1/4 the MIC of the WT strain. |
| DA70127 | This study | *E. coli* mutant isolated from evolution in presence of ciprofloxacin at concentration of 1/4 the MIC of the WT strain. |
| DA70128 | This study | *E. coli* mutant isolated from evolution in presence of ciprofloxacin at concentration of 1/4 the MIC of the WT strain. |
| DA70129 | This study | *E. coli* mutant isolated from evolution in presence of ciprofloxacin at concentration of 1/4 the MIC of the WT strain. |
| DA70130 | This study | *E. coli* mutant isolated from evolution in presence of ciprofloxacin at concentration of 1/4 the MIC of the WT strain. |
| DA70132 | This study | *E. coli* mutant isolated from evolution in presence of ciprofloxacin at concentration of 1/4 the MIC of the WT strain. |
| DA70133 | This study | *E. coli* mutant isolated from evolution in presence of ciprofloxacin at concentration of 1/10 the MIC of the WT strain. |
| DA70134 | This study | *E. coli* mutant isolated from evolution in presence of ciprofloxacin at concentration of 1/10 the MIC of the WT strain. |
| DA70135 | This study | *E. coli* mutant isolated from evolution in presence of ciprofloxacin at concentration of 1/10 the MIC of the WT strain. |
| DA70136 | This study | *E. coli* mutant isolated from evolution in presence of ciprofloxacin at concentration of 1/10 the MIC of the WT strain. |
| DA70137 | This study | *E. coli* mutant isolated from evolution in presence of ciprofloxacin at concentration of 1/10 the MIC of the WT strain. |
| DA70138 | This study | *E. coli* mutant isolated from evolution in presence of ciprofloxacin at concentration of 1/10 the MIC of the WT strain. |

**Supplementarty Table 2**. Frequency of resistant mutants observed at different sub-MICs

| Fosfomycin | Selective Concentration | 2xMIC | 8xMIC | 16xMIC |
|---|---|---|---|---|
| | 1/4 | 1 | 1 | 1 |
| | 1/10 | 1 | 1 | 1 |
| | 1/20 | 1 | 1 | 1 |
| | 1/50 | 1 | 1 | 1 |
| | 1/200 | 2.90E-04 | 1.60E-04 | 2.80E-05 |
| | 1/500 | 2.80E-04 | 3.40E-05 | 8.90E-06 |
| | 1/1000 | 1.80E-04 | 1.30E-05 | 1.10E-05 |
| | 1/2000 | 2.00E-04 | 4.00E-05 | 6.20E-06 |
| | control evolved populations | 1.19E-05 | 4.39E-06 | 1.76E-06 |

| Tetracycline | Selective Concentration | 4X | 8X | 16X |
|---|---|---|---|---|
| | 1/4 | 1 | 1 | 1 |
| | 1/10 | 1 | 1 | 2.80E-06 |
| | 1/20 | 1 | 1 | 5.50E-05 |
| | 1/50 | 1 | 9.90E-05 | 3.90E-06 |
| | 1/200 | 1 | 9.70E-06 | 0 |
| | 1/500 | 4.90E-06 | 0 | 0 |
| | 1/1000 | 6.50E-06 | 0 | 0 |
| | 1/2000 | 6.50E-06 | 0 | 0 |
| | control evolved populations | 1.6E-05 | 0 | 0 |

| Nitrofurantoin | Selective Concentration | 4xMIC | 8xMIC | 16xMIC |
|---|---|---|---|---|
| | 1/4 | 1 | 1.8 x 10-6 | 5.0 x 10-7 |
| | 1/10 | 1 | 5.0 x 10-7 | 0 |
| | 1/20 | 4.10E-05 | 6.2 x 10-7 | 0 |
| | 1/50 | 7.20E-05 | 5.0 x 10-7 | 0 |
| | 1/200 | 1 | 1.0 x 10-5 | 0 |
| | 1/500 | 1.00E-05 | 0 | 0 |
| | 1/1000 | 2.00E-05 | 0 | 0 |
| | 1/2000 | 3.80E-06 | 0 | 0 |
| | control evolved populations | 3.21E-07 | 0 | 0 |

| Ciprofloxacin | Selective Concentration | 2xMIC | 8xMIC | 16xMIC |
|---|---|---|---|---|
| | 1/4 | 1 | 1 | 0 |
| | 1/10 | 1 | 0 | 0 |
| | 1/20 | 0 | 0 | 0 |
| | 1/50 | 0 | 0 | 0 |
| | 1/200 | 0 | 0 | 0 |
| | 1/500 | 0 | 0 | 0 |
| | 1/1000 | 0 | 0 | 0 |
| | 1/2000 | 0 | 0 | 0 |
| | control evolved populations | 0 | 0 | 0 |

**Supplementary Table 3**. Description of genes that contain mutations as identified by whole-genome sequencing of resistant mutants.

| Gene | Description | Clones with mutations (Mutation) | Potential fitness benefit |
|---|---|---|---|
| *aat* | leucyl/phenylalanyl-tRNA-protein transferase | DA66928 (G146D); DA67610 (N191D); DA67619 (N191D); DA67620 (N191D); DA68060 (c.125::IS1); DA68063 (c.125::IS1) | Undetermined |
| *allB* | allantoinase | DA66935 (S45R) | Undetermined |
| *bglG* | transcriptional anti terminator | DA66931 (L82*) | Media adaptation[1] |
| *brnQ* | membrane protein | DA70124 (ΔM419-L422) | Undetermined |
| *caiD* | crotonobetainyl-CoA hydratase | DA70123 (E167A) | Undetermined |
| *cheA* | histidine protein kinase | DA68058 (A42V); DA68062 (A42V) | Undetermined |
| *cho* | endonuclease of nucleotide excision repair | DA70135 (A131T) | Undetermined |
| *degQ* | periplasmic serine endoprotease | DA68062 (I119T) | Antibiotic resistance[2] |
| *dhaK* | dihydroxyacetone kinase subunit K | DA67608 (G268=) | Undetermined |
| *elaD* | deubiquitinating protease | DA67607 (A321=) | Undetermined |
| *emrA* | multidrug efflux pump membrane fusion protein | DA70121 (P197=) | Antibiotic resistance[3] |
| *envZ* | sensor histidine kinase | DA67599 (L22F); DA67601 (R14C); DA67602 (R14C); DA67604 (P148S); DA67608 (R397C); DA67609 (R397C) DA67610 (V49A); DA67619 (V49A); DA67620 (V49A) | Antibiotic resistance[4,5] |
| *fbaA* | fructose-bisphosphate aldolase | DA67599 (N154S); DA67602 (V235A) | Antibiotic resistance[2] |
| *fimE* | fimbral protein | DA66920 (c.214::IS1); DA66922 (c.125::IS5); DA66923 (c.125::IS5); DA66925 (c.272::IS5) DA66927 (c.272::IS5); DA66929 (c.125::IS5); DA66930 (c.125::IS5) DA66935 (c.201::IS1); DA67602 (c.501::IS1); DA67604 (c.168::IS1) DA67605 (c.272::IS5); DA67608 (c.272::IS5); DA67609 (c.272::IS5) DA67610 (c.272::IS5); DA67612 (c.125::IS5); DA67618 (c.125::IS5) DA67619 (c.125::IS5); DA67620 (c.125::IS5) ; DA67621 (c.125::IS5) DA68057 (c.272::IS5); DA68058 (T87P); DA68060 (H73P) DA68062 (T87P); DA68063 (H73P); DA68074 (c.125::IS5) DA69423 (c.125::IS5); DA69424 (c.6::IS1) ; DA69427 (G163 fs) DA69428 (G163 fs); DA69429 (c.519::IS5) ; DA69430 (c.519::IS5) DA70123 (c.125::IS5); DA70129 (c.125::IS5); DA70130 (c.125::IS5) DA70132 (c.125::IS5) | Media adaptation[6] |
| *gatA* | galactitol-specific PTS enzyme | DA69427 (c.203::IS5) | Undetermined |
| *gatB* | galactitol-specific PTS enzyme | DA69428 (c.68::IS5) | Undetermined |
| *gatC* | galactitol-specific PTS enzyme | DA67605 (c.85::IS5); DA67608 (c.85::IS5); DA67609 (c.85::IS5) DA67610 (c.626::IS5); DA67612 (c.626::IS5); DA67619 (c.626::IS5) DA67620 (c.626::IS5); DA67621 (c.626::IS5) | Undetermined |
| *gatY* | tagatose-1,6-bisphosphate aldolase 2 | DA67599 (c.644::IS5) | Media adaptation[6] |
| *glpT* | *sn*-glycerol 3-phosphate: phosphate antiporter | DA66920 (D88E); DA66921 (c.862::IS5); DA66922 (c.862::IS5) DA66923 (c.862::IS5); DA66924 (V86 fs); DA66925 (D88E) DA66926 (L255R); DA66927 (D88E); DA66933 (P212 fs) | Antibiotic resistance[7] |

| | | | |
|---|---|---|---|
| | | DA69421 (c.909::IS5); DA69429 (Δ I443-R456) | |
| *gyrA* | DNA gyrase subunit A | DA70123 (G81D); DA70124 (S96 fs); DA70129 (G81D) DA70130 (G81D); DA70132 (G81D); DA70135 (D40 fs) DA70137 (D87G) | Antibiotic resistance[8] |
| *gyrB* | DNA gyrase subunit B | DA70121 (A65 fs), DA70122 (A65 fs) DA70133 (A65 fs); DA70133 (A65 fs) | Antibiotic resistance[9,10] |
| *hchA* | protein/nucleic acid deglycase 1 | DA66929 (c.635::IS1); DA66930 (c.635::IS1) | Undetermined |
| *icd* | isocitrate dehydrogenase | DA70121 (H366=; L375=) DA70122 (H336=; T370=; L375=; N385=; A366=; K387=) DA70133 (H366=; L375=, T370=); DA70133 (H366=; L375=, T370=) | Undetermined |
| *iscR* | DNA-binding transcriptional dual regulator | DA666924 (P61L) | Undetermined |
| *kdpD* | sensor histidine kinase | DA66921 (R508H); DA66922 (D774G); DA66923 (D774G) DA66924 (G836E); DA66926 (R620L); DA66929 (R511L) DA66930 (R511L); DA66931 (E651A); DA67610 (S275P) DA67613 (V329A); DA67620 (S275); DA68056 (V845G) DA68059 (S541P); DA68060 (A426V); DA68063 (A426V) DA68064 (T633M); DA69427 (R508H); DA69428 (R508H) DA70133 (R508H); DA70134 (R508H) | Media adaptation (this study) |
| *leuV* | tRNA-Leu (CAG) | DA68069 (c.65::IS186) | Undetermined |
| *malT* | DNA-binding transcriptional activator | DA67599 (T220P) | Media adaptation[6] |
| *mdtF* | multidrug efflux pump permease | DA66921 (c.1402::IS1) DA66929 (c.1402::IS1); DA66930 (c.1402::IS1) DA66935 (c.1402::IS1); DA67599 (c.402::IS1) DADA67610 (V766A); DA67611 (c.1402::IS1) DA67613 (c.1402::IS1) | Antibiotic resistance[11] |
| *mdtH* | multidrug efflux pump | DA68062 (P99Q) | Antibiotic resistance[12] |
| *menC* | o-succinylbenzoate synthase | DA68069 (c.132::IS196) | Undetermined |
| *mpaA* | murein tripeptide amidase A | DA68056 (D185E) | Undetermined |
| *mutL* | DNA mismatch repair protein | DA67610 (L92P); DA67612 (L92P); DA67618 (L92P) DA67619 (L92P); DA67620 (L92P) | Undetermined |
| *mutS* | DNA mismatch repair protein | DA67601 (G218E; c.2444::IS1); DA67602 (c.2444::IS1) DA67613 (R656S) | Undetermined |
| *mutT* | 8-oxo-dGTP diphosphatase | DA68057 (R23L) | Undetermined |
| *nfsA* | NADPH-dependent nitroreductase/ NADPH-dependent quinone reductase | DA68056 (L98 fs); DA68057(L145*); DA68058 (W212*) DA68062 (c.380::IS186); DA68063 (c.380::IS186) DA68064 (c.380::IS186); DA68067 (c.674::IS30) DA68068 (c.674::IS30); DA68069 (c.380::IS186) DA68070 (c.674::IS30); DA68071 (c.674::IS30) DA68072 (c.380::IS186); DA68074 (c.380::IS186) DA68076 (c.674::IS30) | Antibiotic resistance[13] |
| *nfsB* | NAD(P)H-dependent nitroreductase | DA68058 (c.38::IS5); DA68069 (c.38::IS5) DA68072 (c.38::IS5); DA68074 (c.38::IS5) | Antibiotic resistance[13] |
| *nhaR* | DNA-binding transcriptional activator | DA69424 (ΔI124-C360 IS1-mediated) | Undetermined |
| *nmpC* | DLP12 prophase; putative outer membrane porin | DA66926 (ΔL1-A337 IS5-mediated) | Undetermined |
| *oxyR* | DNA-binding transcriptional dual regulator | DA70135 (L191 fs) | Undetermined |
| *plsB* | glycerol-3-phosphate 1-O-acyltransferase | DA67608 (R265L); DA67609 (R265L) | Undetermined |
| *proB* | glutamate 5-kinase | DA70130 (R118 fs); DA70131 (R118 fs) | Undetermined |
| *ptsI* | phosphoenolpyruvate-protein phosphotransferase PtsI | DA66934 (H439 fs) | Antibiotic resistance[14] |

| | | | |
|---|---|---|---|
| *ptsP* | phosphoenolpyruvate-protein phosphotransferase PtsP | DA66920 (Q340*); DA66932 (A239 fs); DA66933 (A239 fs)<br>DA66934 (A239 fs); DA67610 (C668R); DA67612 (C668R)<br>DA67613 (S355P); DA67619 (C668R); DA67620 (C668R)<br>DA68057 (L425*); DA68058 (N23 fs); DA68062 (N23 fs)<br>DA70121 (R119 fs), DA70123 (R119 fs); DA70124 (R119 fs)<br>DA70129 (L111 fs); DA70130 (L111 fs); DA70132 (L111 fs)<br>DA70135 (T560 fs) | Media adaptation |
| *ptwF* | | DA68060 (R1=); DA68063 (R1=); DA68069 (R1=) | Undetermined |
| *putP* | proline:$Na^+$ symporter | DA66935 (L60Q); DA68060 (V338L); DA68063 (V338L) | Undetermined |
| *puuB* | γ-glutamylputrescine oxidase | DA69424 (G254=) | Undetermined |
| *rbsR* | DNA-binding transcriptional dual regulator | DA66928 (A181T); DA66932 (c.360::IS2); DA66933 (c.360::IS2)<br>DA66934 (c.360::IS2); DA69429 (T169M); DA69430 (T169M) | Undetermined |
| *relA* | GDP/GTP pyrophosphokinase | DA68067 (Δ A274-G727); DA68068 (Δ A274-G727)<br>DA68070 (Δ A274-G727); DA68071 (Δ A274-G727)<br>DA68076 (Δ A274-G727) | Media adaptation[6] |
| *rho* | transcription termination factor | DA67599 (R87C); DA67604 (S84C); DA68064 (R102S) | Antibiotic resistance[15] |
| *rph* | truncated RNAase PH | DA66921 (E507*); DA66928 (ΔA203-R230); DA68056 (ΔA203-R230)<br>DA68059 (ΔA203-R230); DA68060 (ΔA203-R230)<br>DA68063 (ΔA203-R230); DA69421 (ΔA203-R230)<br>DA69423 (ΔA203-R230); DA69424 (ΔA203-R230)<br>DA69429 (ΔA203-R230); DA69430 (ΔA203-R230)<br>DA70122 (S186 fs); DA70135 (ΔA203-R230) | Media adaptation[6] |
| *rpoC* | RNA polymerase subunit β' | DA67601 (A726T; T1050A) | Media adaptation[6] |
| *rpsF* | 30S ribosomal subunit protein S6 | DA70137 (A116 fs) | Undetermined |
| *rpsK* | 30S ribosomal subunit protein S11<br>30S ribosomal subunit protein S11 | DA68069 (G39D) | Undetermined |
| *sapC* | putrescine ABC exporter membrane protein SapC | DA69424 (P65T) | Undetermined |
| *ssuA* | aliphatic sulfonate ABC transporter periplasmic binding protein | DA70137 (D337G) | Undetermined |
| *ssuD* | $FMNH_2$-dependent alkanesulfonate monooxygenase | DA70122 (E243=) | Undetermined |
| *suhB* | Nus factor SuhB | DA67618 (G233=) | Undetermined |
| *tap* | methyl-accepting chemotaxis protein-dipeptide-sensing protein | DA70129 (c.822_832dup) | Undetermined |
| *trkA* | NAD-binding component of Trk potassium transporters | DA67608 (S360R) | Media adaptation[6] |
| *trkH* | K+ transporter TrkH | DA66925 (L80K); DA66927 (L80K)<br>DA66935 (Y130H); DA70122 (A347D) | Media adaptation and Antibiotic resistance[6,16] |
| *ubiF* | 2-octaprenyl-3-methyl-6-methoxy-1,4-benzoquinol hydroxylase | DA66922 (R235=); DA66923 (R235=) | Antibiotic resistance[17] |
| *uhpA* | DNA-binding transcriptional activator | DA66924 (ΔL43-G45); DA69421 (ΔA41-L43)<br>DA69429 (ΔA41-L43); DA69430 (ΔA41-L43) | Antibiotic resistance[14] |
| *uhpB* | sensory histidine kinase | DA66931 (V33 fs); DA66932 (W132*)<br>DA66933 (G469R); DA66935 (c.1012::IS1) | Antibiotic resistance[18] |
| *uhpC* | inner membrane protein sensing glucose-6-phosphate | DA66922 (T72P); DA6923 (T72P)<br>DA66929 (c.235::IS5); DA66930 (A129T); DA66934 (Q34*) | Antibiotic resistance[18] |

| | | | |
|---|---|---|---|
| | | DA69423 (c.760::IS5); DA69427 (N164K); DA69428 (ΔQ131-S143) | |
| *uhpT* | hexose-6-phosphate:phosphate antiporter | DA66920 (c.214::IS1); DA66921 (F365L); DA66925 (c.948::IS186)<br>DA66927 (c.948::IS186); DA66928 (P423 fs)<br>DA68067 (ΔA335-G427); DA68068 (ΔA335-G427)<br>DA68070 (ΔA335-G427); DA68071 (ΔA335-G427)<br>DA68076 (ΔA335-G427); DA69420 (c.382::IS2)<br>DA69424 (c.118::IS1) | Antibiotic resistance[7,18] |
| *yahL* | uncharacterized protein YahL | DA67599 (c.527::IS5) | Undetermined |
| *ycgB* | PF04293 family protein | DA68069 (c.192::IS2) | Undetermined |
| *ydcI* | DNA-binding transcriptional regulator | DA66921 (c.21::IS1); DA66922 (K258*); DA66923 (c.85::IS2)<br>DA66924 (H77R); DA66927 (T233M); DA66929 (Q58 fs)<br>DA66930 (Q58 fs); DA66931 (F2 fs); DA66933 (c. 241::IS2);<br>DA68056 (N4 fs); DA68064 (c.278::IS2)<br>DA69421 (R14L); DA69423 (L123S); DA69428 (S92 fs)<br>DA70121 (ΔD286-I298); DA70124 (ΔE79-Y248) | Antibiotic resistance[19] |
| *ydcT* | putative ABC transporter ATP-binding protein | DA70133 (S11=) | Undetermined |
| *ydeJ* | PF02464 family protein | DA66933 (c.29::IS2) | Undetermined |
| *ydeO* | DNA-binding transcriptional dual regulator | DA70135 (S2=) | Undetermined |
| *ydhL* | DUF1289 domain-containing protein | DA70124 (R63H) | Undetermined |
| *yeaI* | putative c-di-GMP binding protein | DA66924 (F343L) | Undetermined |
| *yeaR* | DU1971 domain-containing protein | DA67599 (c.115::IS186); DA68067 (c.115::IS186)<br>DA68068 (c.115::IS186); DA68070 (c.115::IS186)<br>DA68074 (c.115::IS186); DA68076 (c.115::IS186) | Undetermined |
| *yegX* | putative glycosyl hydrolase | DA69420 (P192Q) | Undetermined |
| *yehH* | pseudogene | DA66923 (V254A) | Undetermined |
| *yfaS* | putative macroglobulin family protein, N-terminal family | DA68068 (T30I) | Undetermined |
| *yfdE* | acetyl-CoA:oxalate CoA-transferase | DA68058 (c.457::IS2) | Undetermined |
| *yffS* | CPZ-55 prophase; uncharacterized protein YffS | DA66928 (A148T) | Undetermined |
| *yhaC* | uncharacterized protein | DA68056 (c.1101::IS5) | Undetermined |
| *yhaI* | putative inner membrane protein | DA70122 (c.68::IS1) | Undetermined |
| *ymdA* | uncharacterized protein | DA66930 (G59 fs); DA66933 (c.289::IS2); DA67610 (c.303::IS5);<br>DA67611 (c.48delT); DA67612 (c.309::IS5); DA67613 (c.309::IS5)<br>DA67618 (c.309::IS5); DA67619 (c.309::IS5); DA67620 (c.309::IS5)<br>DA67621 (c.309::IS5); DA70123 (c.308::IS5); DA70129 (F18 fs); DA70130 (c.309::IS5); DA70132 (c.309::IS5) | Undetermined |
| *yobF* | DUF2527 domain-containing protein | DA68059 (c.106::IS1); DA69427 (R19P) | Undetermined |
| *yphG* | UF5107 domain-containing protein | DA69427 (L750=); DA69428 (L750=)<br>DA70121 (L750=); DA70122 (L750=)<br>DA70133 (L750=); DA70134 (L750=) | Undetermined |

## References:


1. Madan, R., Kolter, R. & Mahadevan, S. Mutations that activate the silent bgl operon of Escherichia coli confer a growth advantage in stationary phase. *J Bacteriol* **187**, 7912–7917 (2005).
2. Gu, Y. *et al.* Development of Resistance in Escherichia coli ATCC25922 under Exposure of Sub-Inhibitory Concentration of Olaquindox. *Antibiotics (Basel)* **9**, E791 (2020).
3. Lomovskaya, O., Lewis, K. & Matin, A. EmrR is a negative regulator of the Escherichia coli multidrug resistance pump EmrAB. *J Bacteriol* **177**, 2328–2334 (1995).
4. Adler, M., Anjum, M., Andersson, D. I. & Sandegren, L. Combinations of mutations in envZ, ftsI, mrdA, acrB and acrR can cause high-level carbapenem resistance in Escherichia coli. *J Antimicrob Chemother* **71**, 1188–1198 (2016).
5. Jordan, J. A., Lenski, R. E. & Card, K. J. Idiosyncratic Fitness Costs of Ampicillin-Resistant Mutants Derived from a Long-Term Experiment with Escherichia coli. *Antibiotics (Basel)* **11**, 347 (2022).
6. Knöppel, A. *et al.* Genetic Adaptation to Growth Under Laboratory Conditions in Escherichia coli and Salmonella enterica. *Front Microbiol* **9**, 756 (2018).
7. Fu, Z. *et al.* Prevalence of Fosfomycin Resistance and Mutations in murA, glpT, and uhpT in Methicillin-Resistant Staphylococcus aureus Strains Isolated from Blood and Cerebrospinal Fluid Samples. *Front Microbiol* **6**, 1544 (2015).
8. Lee, J. K., Lee, Y. S., Park, Y. K. & Kim, B. S. Mutations in the gyrA and parC genes in ciprofloxacin-resistant clinical isolates of Acinetobacter baumannii in Korea. *Microbiol Immunol* **49**, 647–653 (2005).
9. Feng, X. *et al.* Mutations in gyrB play an important role in ciprofloxacin-resistant Pseudomonas aeruginosa. *Infect Drug Resist* **12**, 261–272 (2019).
10. Gensberg, K., Jin, Y. F. & Piddock, L. J. A novel gyrB mutation in a fluoroquinolone-resistant clinical isolate of Salmonella typhimurium. *FEMS Microbiol Lett* **132**, 57–60 (1995).
11. Bohnert, J. A., Schuster, S., Fähnrich, E., Trittler, R. & Kern, W. V. Altered spectrum of multidrug resistance associated with a single point mutation in the Escherichia coli RND-type MDR efflux pump YhiV (MdtF). *J Antimicrob Chemother* **59**, 1216–1222 (2007).
12. Rani, A. *et al.* Abundance of Multidrug Resistance Efflux Pumps in the Urinary Metagenome of Kidney Transplant Patients. *Biomed Res Int* **2020**, 5421269 (2020).
13. Wan, Y. *et al.* Alterations in chromosomal genes nfsA, nfsB, and ribE are associated with nitrofurantoin resistance in Escherichia coli from the United Kingdom. *Microb Genom* **7**, (2021).
14. Silver, L. L. Fosfomycin: Mechanism and Resistance. *Cold Spring Harb Perspect Med* **7**, a025262 (2017).
15. Lee, Y. H. & Helmann, J. D. Mutations in the primary sigma factor σA and termination factor rho that reduce susceptibility to cell wall antibiotics. *J Bacteriol* **196**, 3700–3711 (2014).
16. Apjok, G. *et al.* Limited Evolutionary Conservation of the Phenotypic Effects of Antibiotic Resistance Mutations. *Mol Biol Evol* **36**, 1601–1611 (2019).
17. Pinheiro, F., Warsi, O., Andersson, D. I. & Lässig, M. Metabolic fitness landscapes predict the evolution of antibiotic resistance. *Nat Ecol Evol* **5**, 677–687 (2021).
18. Silver, L. L. Fosfomycin: Mechanism and Resistance. *Cold Spring Harb Perspect Med* **7**, a025262 (2017).
19. Hingley-Wilson, S. M. *et al.* Loss of phenotypic inheritance associated with ydcI mutation leads to increased frequency of small, slow persisters in Escherichia coli. *Proc Natl Acad Sci U S A* **117**, 4152–4157 (2020).

**Supplementary table 4:** Cross-resistance tested for subset of mutants selected on tetracycline and nitrofurantoin

| Isolate | Genotype | Selection | Cross-resistance tested on | | | |
|---|---|---|---|---|---|---|
| | | | FOS | TET | NIT | CIP |
| DA5438 | - | - | 0.38 | 1.5 | 4 | 0.012 |
| DA67604 | *envZ* (P148S); *rho* (S84C); *fimE* (c.501::IS1) | TET (1/4x MIC) | 1 | | 8 | 0.38 |
| DA68067 | *uhpT* (A335-G427del); *relA* (A274-G727); *nfsA* (c.674::IS30); *yeaR* (c.115::IS186) | NIT (1/4x MIC) | 128 | 1.5 | | 0.008 |
| DA68068 | *uhpT* (A335-G427del); *relA* (A274-G727); *nfsA* (c.674::IS30); *yeaR* (c.115::IS186); *yfaS* (R30I) | NIT (1/4x MIC) | 64 | 1 | | 0.016 |
| DA68070 | *uhpT* (A335-G427del); *relA* (A274-G727); *nfsA* (c.674::IS30); *yeaR* (c.115::IS186) | NIT (1/4x MIC) | 64 | 1.5 | | 0.012 |
| DA68071 | *uhpT* (A335-G427del); *relA* (A274-G727); *nfsA* (c.674::IS30) | NIT (1/4x MIC) | 48 | 0.75 | | 0.012 |
| DA68076 | *uhpT* (A335-G427del); *relA* (A274-G727); *nfsA* (c.674::IS30); *yeaR* (c.115::IS186) | NIT (1/4x MIC) | 32-64 | 1 | | 0.012 |
| DA68060 | *ptwF* (R1=); *putP* (V338L); *aat* (c.125::IS1); Δ *yrdA-yrdD* (562bp); *kdpD* (A426V); *rpH* (A203-R230del); *fimE* (H73P) | NIT (1/200x MIC) | 0.75 | 1 | | 0.006 |
| DA68063 | *nfsA* (c.380::IS186); *ptwF* (R1=); *putP* (V338L); *aat* (c.125::IS1); *kdpD* (A426V); *rpH* (A203-R230del); *fimE* (H73P) | NIT (1/200x MIC) | 1.5 | 1.5 | | 0.008 |
